# A modelling study to dissect the potential role of voltage-gated ion channels in activity-dependent conduction velocity changes as identified in small fiber neuropathy patients

**DOI:** 10.3389/fncom.2023.1265958

**Published:** 2023-12-14

**Authors:** Anna Maxion, Ekaterina Kutafina, Maike F. Dohrn, Pierre Sacré, Angelika Lampert, Jenny Tigerholm, Barbara Namer

**Affiliations:** ^1^Research Group Neuroscience, Interdisciplinary Centre for Clinical Research within the Faculty of Medicine at the RWTH Aachen University, Aachen, Germany; ^2^Institute of Medical Informatics, Medical Faculty, RWTH Aachen University, Aachen, Germany; ^3^Department of Neurology, Medical Faculty, RWTH Aachen University, Aachen, Germany; ^4^Department of Electrical Engineering and Computer Science, University of Liège, Liège, Belgium; ^5^Institute of Neurophysiology, Uniklinik RWTH Aachen University Aachen, Aachen, Germany; ^6^Joint Research Center for Computational Biomedicine, RWTH Aachen, Aachen, Germany; ^7^Institute of Neurophysiology, RWTH Aachen University, Aachen, Germany; ^8^Institute of Physiology and Pathophysiology, University of Erlangen-Nürnberg, Erlangen, Germany

**Keywords:** neuron, simulation, C-fiber axon, neuropathic pain, microneurography, voltage-gated ion channels, in-silico model

## Abstract

**Objective:**

Patients with small fiber neuropathy (SFN) suffer from neuropathic pain, which is still a therapeutic problem. Changed activation patterns of mechano-insensitive peripheral nerve fibers (CMi) could cause neuropathic pain. However, there is sparse knowledge about mechanisms leading to CMi dysfunction since it is difficult to dissect specific molecular mechanisms in humans. We used an *in-silico* model to elucidate molecular causes of CMi dysfunction as observed in single nerve fiber recordings (microneurography) of SFN patients.

**Approach:**

We analyzed microneurography data from 97 CMi-fibers from healthy individuals and 34 of SFN patients to identify activity-dependent changes in conduction velocity. Using the NEURON environment, we adapted a biophysical realistic preexisting CMi-fiber model with ion channels described by Hodgkin-Huxley dynamics for identifying molecular mechanisms leading to those changes. Via a grid search optimization, we assessed the interplay between different ion channels, Na-K-pump, and resting membrane potential.

**Main results:**

Changing a single ion channel conductance, Na-K-pump or membrane potential individually is not sufficient to reproduce in-silico CMi-fiber dysfunction of unchanged activity-dependent conduction velocity slowing and quicker normalization of conduction velocity after stimulation as observed in microneurography. We identified the best combination of mechanisms: increased conductance of potassium delayed-rectifier and decreased conductance of Na-K-pump and depolarized membrane potential. When the membrane potential is unchanged, opposite changes in Na-K-pump and ion channels generate the same effect.

**Significance:**

Our study suggests that not one single mechanism accounts for pain-relevant changes in CMi-fibers, but a combination of mechanisms. A depolarized membrane potential, as previously observed in patients with neuropathic pain, leads to changes in the contribution of ion channels and the Na-K-pump. Thus, when searching for targets for the treatment of neuropathic pain, combinations of several molecules in interplay with the membrane potential should be regarded.

## 1 Introduction

Neuropathic pain affects 7–10% of the population and results from damage to the somatosensory system (Colloca et al., 2017; Petroianu et al., 2023). Factors such as an aging population, diabetes, and cancer treatment increase the incidence of neuropathic pain, which presents itself in a diverse array of manifestations. Small fiber neuropathy (SFN) is a medical condition that affects the small sensory cutaneous nerve fibers, such as Aδ and C-fibers (Sène, 2018; Strand et al., 2022). Common is a mechanically evoked hyperalgesia in the extremities (Serra et al., 2011; Chan and Wilder-Smith, 2016). However, for unknown reasons, not all patients with neuropathy experience pain. Unfortunately, up to now, there is no biomarker for ongoing pain in patients with SFN. Identifying a reliable assessment of excitability alterations in patients is important for understanding the pathophysiological mechanisms underlying neuropathic pain and why some patients develop pain while others do not.

Voltage-gated sodium channels (Na_*v*_) play an important role in pain signaling as shown by monogenic diseases affecting pain sensitivity ranging from insensitivity to pain to extreme painful disorders such as paroxysmal pain disorder (Habib et al., 2015; Bennett et al., 2019; Körner and Lampert, 2020; Goodwin and McMahon, 2021). SFN can be associated with gene variants in the genes of the subtypes Na_*v*_1.7, Na_*v*_1.8, or Na_*v*_1.9 (Huang et al., 2014; Chan and Wilder-Smith, 2016). Thus, the function of voltage-gated sodium channels, especially of the subtypes Na_*v*_1.7, Na_*v*_1.8, and Na_*v*_1.9 are prone candidates to account for differences between patients with or without ongoing neuropathic pain. However, also other membrane properties and molecules such as resting membrane potential (RMP), sodium potassium pump (Na-K-pump), or voltage-gated potassium channels modulate the excitability of the membrane and thus can play a critical role in ongoing neuropathic pain (Burke et al., 2001; Krishnan et al., 2008; Busserolles et al., 2016).

Microneurography allows to assess the conduction of nerve signals (action potentials) in peripheral nerve fibers in healthy human volunteers, as well as in diseased patients with neuropathic pain. With this method, the response of single unmyelinated nerve fibers (C-fibers) to electrical stimuli can be recorded and mechano-sensitive (CM) or mechano-insensitive (CMi) fibers can be identified. In this study, we are focusing on CMi-fibers which become hyperexcitable and spontaneously active in patients with diabetic neuropathy and erythromelalgia correlating to the presence of ongoing pain (Kleggetveit et al., 2012).

During microneurography, the receptive field of the C-fibers is stimulated with electrical, mechanical, or chemical stimuli, which results in the creation of action potentials that travel from the stimulation site to the recording electrode. The corresponding delay between stimulation and recording time is called latency and increases for higher frequencies of electrical stimulation, i.e., the response of the fiber becomes slower and is therefore referred to as activity-dependent slowing (ADS). Reducing the frequency of the stimulation leads to a return of the latency to its baseline level, i.e., normalization of latency (Schmelz et al., 1995; Weidner et al., 1999).

When assessing pain patients using microneurography, several changes in the activity-dependent conduction velocity were found, when compared to healthy volunteers. An erythromelalgia patient with a gain-of-function mutation in $Na_{v}1.7$ (I848T) showed frequency-dependent speeding of conduction velocity instead of slowing, as compared to the healthy control group (Namer et al., 2015). Another erythromelalgia patient with a mutation in $Na_{v}1.8$ exhibited an exaggerated ADS (Kist et al., 2016). In (Namer et al., 2009), healthy elderly volunteers expressed more ADS than younger subjects, as well as a slower normalization of latency after stimulation. However, the normalization of latency is quicker for SFN patients with pain than for those without pain (Kleggetveit et al., 2012).

ADS is enhanced by the slow inactivation of Na_*v*_s (De Col et al., 2008) and intracellular sodium accumulation (Tigerholm et al., 2014). Thus, a faster normalization of latency could indicate that sodium channels emerge faster from slow inactivation, leading to a reduced time until the possible initiation of the next action potential, supporting higher overall excitability of the cell and higher discharge rates (De Col et al., 2012). However, it is unknown which ion channel subtypes or other mechanisms, such as Na-K-pump activity and RMP, contribute to enhanced excitability in neuropathic pain patients.

Due to ethical concerns, certain experimental procedures or pharmacological manipulations cannot be performed in microneurography in humans. Thus, to explore the mechanisms underlying changes in ADS and the normalization processes of ADS computational model systems are needed. The use of animal models is limited since specific types of nerve fibers, such as CMi-fibers, are absent in the skin of rodents (Prato et al., 2017). To overcome these limitations, we used a computational model (Tigerholm et al., 2014) based on microneurographic recordings in humans to simulate the activity of nerve fibers following electrical stimulation to study the mechanisms underlying quicker normalization in pain vs. non-pain SFN patients. We modulated the conductance of the sodium and potassium ion channels, as well as the Na-K-pump and RMP to assess their importance for the normalization of latency.

## 2 Methods

### 2.1 Computational model

We adapted the computational model of a C-fiber described by (Tigerholm et al., 2014). The reproduction of the specific behavior of C-fibers of the model was verified several times by different authors (Petersson et al., 2014; Tigerholm et al., 2015; Pelot et al., 2021). The Hodgkin-Huxley-type biophysical model was originally implemented using the NEURON simulation environment (Hines and Carnevale, 1997) and its built-in interpreter *hoc* with a MATLAB interface for controlling the simulation and plotting the data. We transferred the model to Python (version 3.11.1), which can be utilized as an interpreter for NEURON (Hines et al., 2009). NEURON was used in version 7.8. The code of the model can be accessed at GitHub (Maxion, 2023). One of the main advantages of using Python is the ability to run and evaluate the simulation within the same environment, streamlining the workflow and increasing efficiency. Originally, the multi-compartmental model consisted of a parent axon connected to a thinner branch axon with different diameters and temperatures. To be able to run extensive parameter searches we simplified the model reducing the fiber length to 1 cm with 600 compartments of uniform temperature (37°C) and diameter (1 μm), which lowered computation time by a factor of 10. The model includes three different sodium channels, $Na_{v}\;1.7$ , $Na_{v}\;1.8$ , and $Na_{v}\;1.9$ , each with an activation gate, a fast inactivation and a slow inactivation gate. The channel $Na_{v}\;1.8$ additionally contains an ultra-slow inactivation gate. Further, four potassium channels are included. The delayed rectifier $K_{dr}$ with an activation gate, the channel $K_{f}$ with an activation and inactivation gate, the channel $K_{s}$ with a fast and slow gate that are combined with factors such that the slow effect makes ¼, while the fast effect makes ¾ of the total effect, and the channel $K_{Na}$ with one gate assuming that the opening of the gate is immediately affected by the concentration of sodium within the axon. Additionally, there is a hyperpolarization-activated channel h with a fast and a slow activation gate (also known as HCN). The included sodium and potassium leak currents are essential for maintaining a stable resting membrane potential (RMP) by ensuring that the total inward and outward currents carried by sodium and potassium ions are equal at rest. The Na-K-pump maintains the electrochemical gradient across the cell membrane. Further, the model allows dynamic changes in sodium and potassium concentrations. The underlying differential equations are solved using the variable timestep method.

### 2.2 Recording technique and subjects

**Figure 1:**
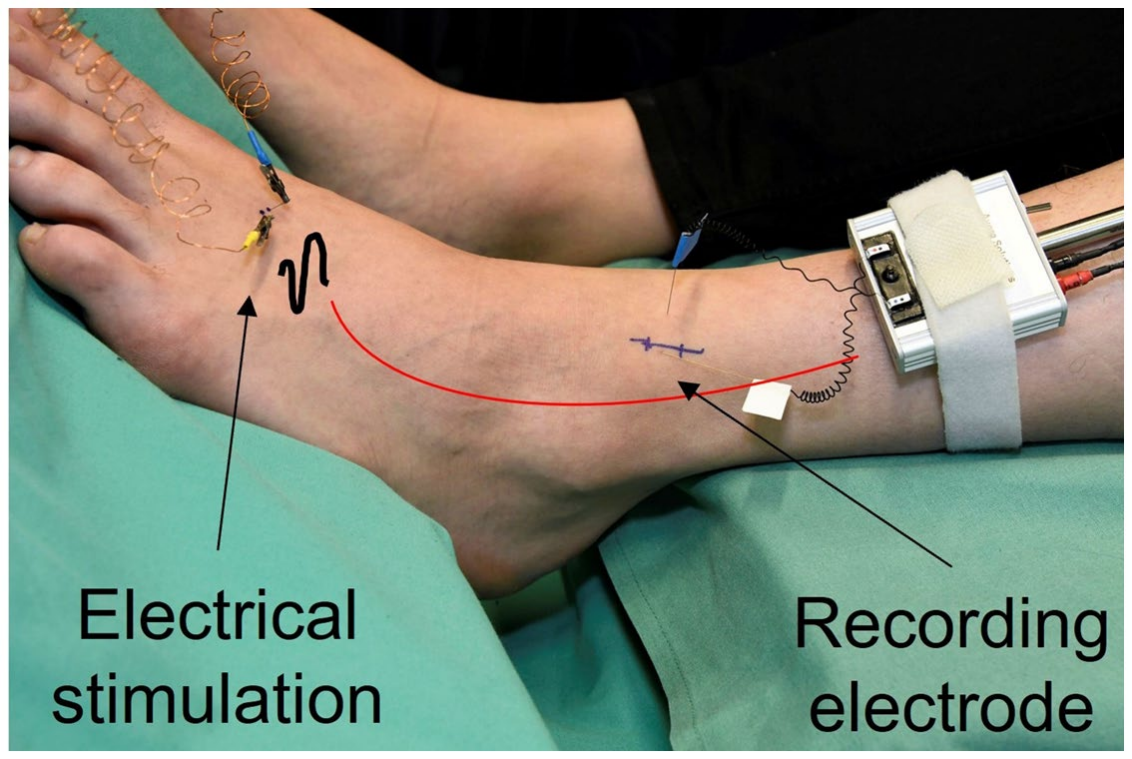
Nerve signals are recorded from single C-fibers. A microelectrode is inserted into the nerve bundle of the superficial peroneal nerve at ankle level (recording electrode). Regular electrical pulses from a constant current stimulator are applied to the receptive field of the fibers, transmitted through superficially inserted electrodes (electrical stimulation).

With the method of microneurography nerve signals are recorded from single C-fibers. An uninsulated microelectrode of 0.005 mm at the tip is inserted into the nerve bundle of the superficial peroneal nerve at ankle level and a reference electrode is situated superficially in the skin close by (Figure 1). Regular electrical pulses, usually ¼ Hz, from an insulated constant current stimulator (Digitimer DS7, Digitimer, Hertfordshire, UK) are applied to the receptive field of the fibers and transmitted through superficially inserted electrodes. This stimulation triggers a response from the fibers in the form of action potentials that occur at a consistent latency, allowing for the identification of multiple fibers within the same recording. Fiber properties such as the mechanical threshold of individual fibers are evaluated using the marking method (Torebjörk and Hallin, 1974; Schmidt et al., 1995). The stimulation as well as the recording of the fiber activity are conducted in DAPSYS (Data Acquisition Processor System (DAPSYS), Brian Turnquist, http://dapsys.net), a system that acquires and processes data in real-time.

Microneurography data, obtained from 97 mechano-insensitive C-fibers (CMi) of healthy individuals, which has been collected and aggregated in our database since 2004, was utilized to validate the representation of CMi-fiber in the computational model. Two fibers had to be excluded from the dataset. One because of incomplete data, due to recording issues and the other fiber was excluded because it was misclassified in the original data set, leading to 95 fibers (Table 1).

Additionally, we examined small fiber neuropathy (SFN) patients in our laboratory to specifically investigate potential differences between patients experiencing pain in the foot or leg and those who did not. The study encompassed 142 nerve fibers from 19 patients in the age range of 23 to 59 years (mean age: 42.8 years), including 11 males and 8 females. For 129 of these fibers the ELID stimulation protocol was performed (Table 1). Some of the fibers showed spontaneous activity, while others did not. Of these fibers, 34 were classified as CMi-fibers according to their receptive properties such as mechanical threshold and response to electrical stimulation. Patients who were classified without pain reported no pain in the foot, where the data was recorded, but experienced pain in other parts of the body. Two fibers were excluded from further analysis, because they showed branching behavior, i.e., one branch of the fiber blocks, which leads the electrical signal being transmitted by another branch of the fiber. This leads to large sudden changes in latency. After excluding incomplete data, 32 CMi-fibers from 13 patients were left. These fibers consist of 9 fibers from 7 patients without pain and 23 fibers from 6 patients with pain at the recording position, i.e., the foot. To compare the different groups statistically, the data sets were checked visually for normal distribution, which was not present. Therefore, a Kruskal-Wallis test with a significance level of α = 0.05 was used to compare healthy individuals and SFN patients with pain and without pain. For the *post hoc* pairwise comparison of the groups, a Wilcoxon rank sum test was utilized. To correct for multiple comparisons, we applied a Bonferroni correction with a significance level of α/3 = 0.017. The statistical analysis was conducted in Python (version 3.11.1).

**Table 1:** Total number of fibers and number of CMi-fibers for the ELID stimulation protocol before and after exclusion of data that did not meet the inclusion criteria, the corresponding number of patients, and the number of males and females for healthy subjects and SFN patients with and without pain.

	Number of all fibers/Number of patients	Number of CMi-fibers/Number of patients	Number of CMi-fibers after exclusion/Number of patients
Healthy	337/-	97/-	95/-
SFN - total	129/19 (m:11, f:8)	34/13 (m:7, f:6)	32/13 (m:7, f:6)
SFN - pain	66/9 (m:4, f:5)	25/6 (m:3, f:3)	23/6 (m:3, f:3)
SFN – no pain	63/10 (m:7, f:3)	9/7 (m:4, f:3)	9/7 (m:4, f:3)

The collection of the data was approved by the local ethics committees from Aachen (EK 141/19) and Erlangen (4361) and conducted in accordance with the principles embodied in the Declaration of Helsinki and statutory requirements of Germany. All participants gave written informed consent to participate in the study.

### 2.3 Stimulation protocol

After a rest period of at least 2 min, the fibers were stimulated with electrical square pulses of 0.5 ms duration of rising frequencies of 1/8 Hz (20 pulses), 1/4 Hz (20 pulses), and 1/2 Hz (30 pulses) and normalization of latency is observed with a frequency of ¼ Hz for at least 10 pulses. The well-known stimulation protocol is used to identify different fiber types, such as mechano-sensitive (CM) and mechano-insensitive (CMi) C-fibers, and therefore called ELID (electrical identification; Weidner et al., 1999).

### 2.4 Defining parameters

**Figure 2:**
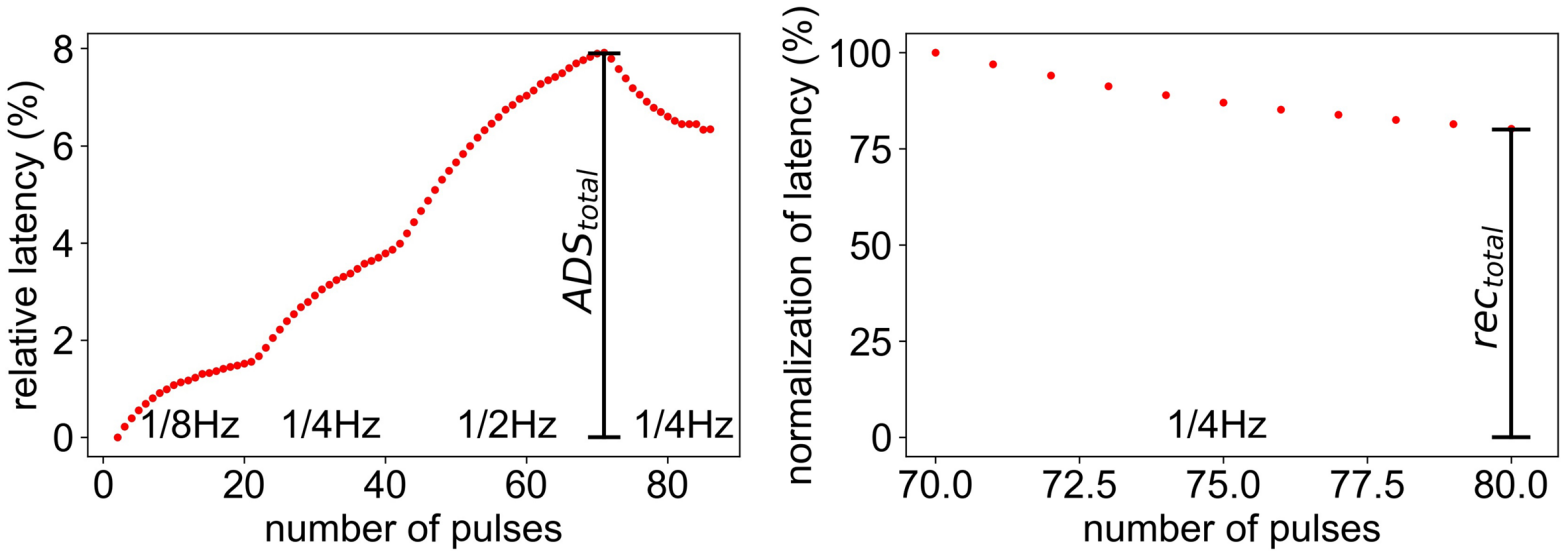
Measurement of $\mathrm{AD}S_{\mathrm{total}}$ and $rec_{\mathrm{total}}$ for the ELID stimulation protocol. (Left) latency relative to initial latency. $\mathrm{AD}S_{\mathrm{total}}$ represents the maximal ADS after high frequency stimulation. (Right) Normalization of latency after the stimulation. The last 10 values of the relative latency (Left) are divided by $\mathrm{AD}S_{\mathrm{total}}$ . $rec_{\mathrm{total}}$ represents the amount of total recovery after 10 pulses during low frequency stimulation.

During ongoing stimulation, the latency, i.e., the time an action potential needs to travel from the point of stimulation to the point of measurement, increases for stimulation with higher frequencies and decreases, if the frequency is lowered. Those changes in response latency are called activity-dependent slowing (ADS). The latency was measured in relation to the initial latency and the maximum ADS ( $\mathrm{AD}S_{\mathrm{total}}$ ) was calculated (Figure 2, left). Additionally, the normalization of latency during stimulation with a lower frequency was measured in relation to the total slowing ( $\mathrm{AD}S_{\mathrm{total}}$ ) to make the normalization comparable for fibers with different total ADS (Figure 2, right). Here, the total recovery ( $rec_{\mathrm{total}}$ ) was used for evaluation.

### 2.5 Method of identifying relevant mechanisms

To implement a computational model that evaluates differences between SFN patients with and without pain, several parameter optimization steps were pursued. The ADS of pain and non-pain patients is similar (pain patients: 7.93% ± 1.36%, non-pain patients: 7.6% ± 0.47%, difference: 0.33%), while the normalization of latency is statistically significantly faster for pain patients (pain patients: 75.8% ± 9.4%, non-pain patients: 84.6% ± 3.2%, difference: −8.8%) for the given protocol (Kleggetveit et al., 2012). An overview of the performed workflow is shown in Figure A1 in the Supplementary Figures and in the following each step is explained in detail.

In the first step, the conductance levels of the ion channels and the Na-K-pump rate, i.e., the conductance of the Na-K-pump, were modulated systematically by increasing and decreasing each level individually by 25% and 50% of its original value independently from each other. The maximal change of 50% was chosen to preserve the original ADS of the fiber. Higher deviations from the original value led to large changes in ADS and could therefore not replicate the desired result. In the model the initial RMP is set at the beginning of each simulation. Changes in the behavior of ion channels, i.e., the conductance, or the Na-K-pump do not affect the initial RMP, unlike in a real fiber. Therefore, the initial RMP was de- and hyperpolarized by 5 mV (original: −55 mV) separately from the other changes to be able to replicate the behavior of a real fiber more accurately. The computational model was evaluated for each individual variation of conductance level, Na-K-pump rate and RMP and the values $\mathrm{AD}S_{\mathrm{total}}$ and $rec_{\mathrm{total}}$ were calculated.

Further, we combined the individual variations of conductance levels, Na-K-pump rate and RMP by adding the values $\mathrm{AD}S_{\mathrm{total}}$ and $rec_{\mathrm{total}}$ for all existing combinations. For example, for a 50% decreased pump rate the model shows an increased value of $\mathrm{AD}S_{\mathrm{total}}$ , while for a depolarized RMP the model shows a decreased value of $\mathrm{AD}S_{\mathrm{total}}$ . If the two models are combined the effects cancel each other out and the $\mathrm{AD}S_{\mathrm{total}}$ in the combined model is near zero. Thus, we could calculate the combined values without needing to evaluate the computational model for each combination. We evaluated which combinations led to a faster normalization of latency, i.e., a smaller value of *rec_total_* by −8.8% ± 0.7%, while simultaneously having only a minor effect on the overall slowing *ADS_total_* of 0.33% ± 0.7%. The 0.7% was added as an error margin. From the resulting combinations, we chose the ones where three or less mechanisms were modulated and executed the model with these parameters. In the resulting simulation data, we measured again ADS and normalization of latency and compared them for each parameter set. We chose the parameter sets where the absolute difference in ADS was below 1% and the difference in normalization of latency was in the interval of [−8.8–4.5%, −8.8 + 4.5%], since the normalization showed a larger variation in the data of SFN patients.

### 2.6 Grid search

Further, we used an algorithm for parameter optimization based on grid search. This method exhaustively tries every combination of the specified parameter values, i.e., conduction of ion channel, Na-K-pump rate, and RMP, in a predefined grid. We chose a grid of five values for the ion channels and the Na-K-pump (−50, −25%, original, +25%, +50%) and three values for the RMP (−5 mV, original, +5 mV) and evaluated the computational model at each point. This allowed us to explore a wide range of possible combinations of conductance values for the selected mechanisms. To reduce the runtime of the grid search, we selected three mechanisms for each run that showed the desired behavior in the previous analysis. The performance of each model was evaluated by comparing the $\mathrm{AD}S_{\mathrm{total}}$ and the normalization of latency $rec_{\mathrm{total}}$ with the desired values of 0.33 for ADS and −8.8% for normalization of latency. To ensure that the perturbation of parameters results in a valid model that preserves the original properties of the in-silico fiber, the spike shape, gating variables and the ionic currents were reviewed visually. For each grid search the model that was closest to these values was chosen. Lastly, the model was run with the optimized parameter sets to verify the result.

## 3 Results

### 3.1 The adapted model resembles CMi-fibers in activity-dependent conduction velocity changes

**Figure 3:**
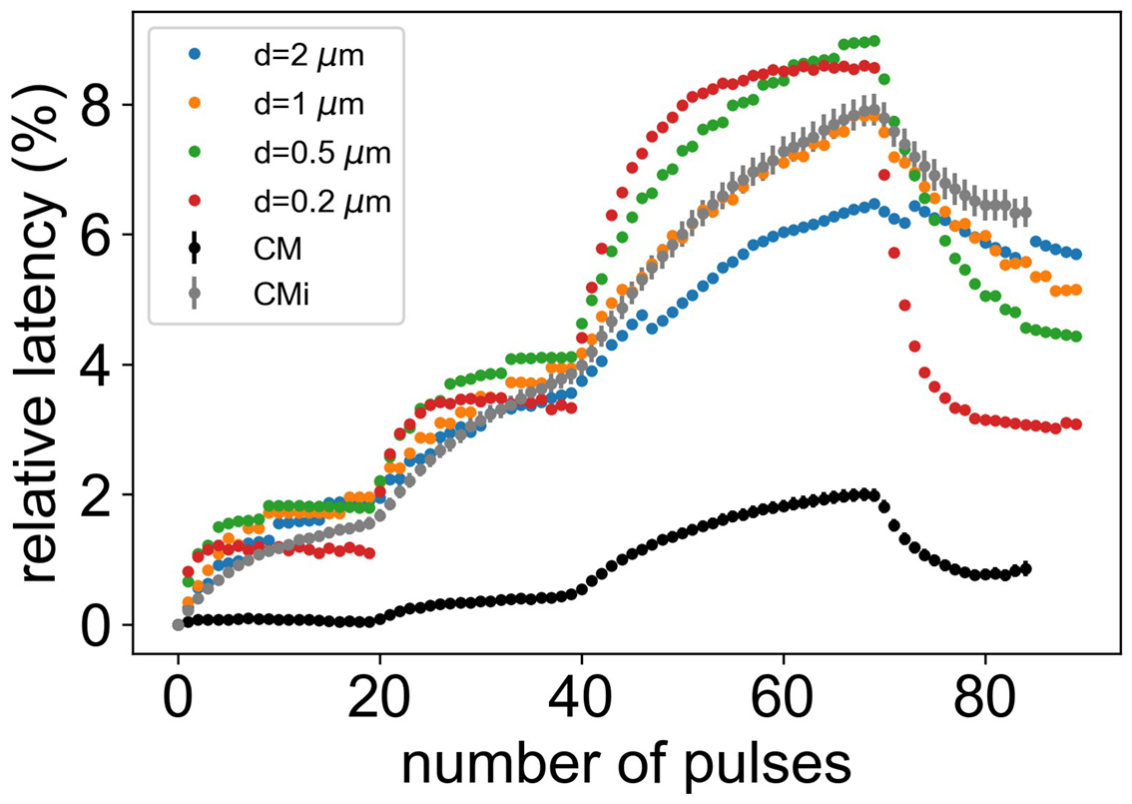
Comparison of microneurography data for CM- and CMi-fibers from healthy subjects with simulation data for different diameters. The error bars show the standard error of the mean. The CM-fiber is added for comparison to provide a basis for better judgment of the variance of different fiber diameters.

To calibrate our computer model to mimic the behavior of healthy human CMi-fibers, we analyzed data recorded from 95 CMi-fibers from healthy subjects using the ELID frequency protocol (indicated by grey markers in Figure 3). In Figure 3, we included 85 CM-fibers (represented by black markers) for comparison purposes, highlighting the more pronounced ADS of the CMi-fibers within this large dataset. In a first step, we aim to determine the neurite diameter of the simulated nerve fibers which produces ADS best fitting to CMi-fibers. We compared the simulated data from the presented computational model (see methods) for four diameters with the data collected from human healthy subjects (Figure 3). For a diameter of 1 μm, the result of the computational model is consistent with electrically stimulated conduction velocity changes of CMi-fibers of healthy volunteers.

### 3.2 Normalization of latency is faster for pain patients

**Figure 4:**
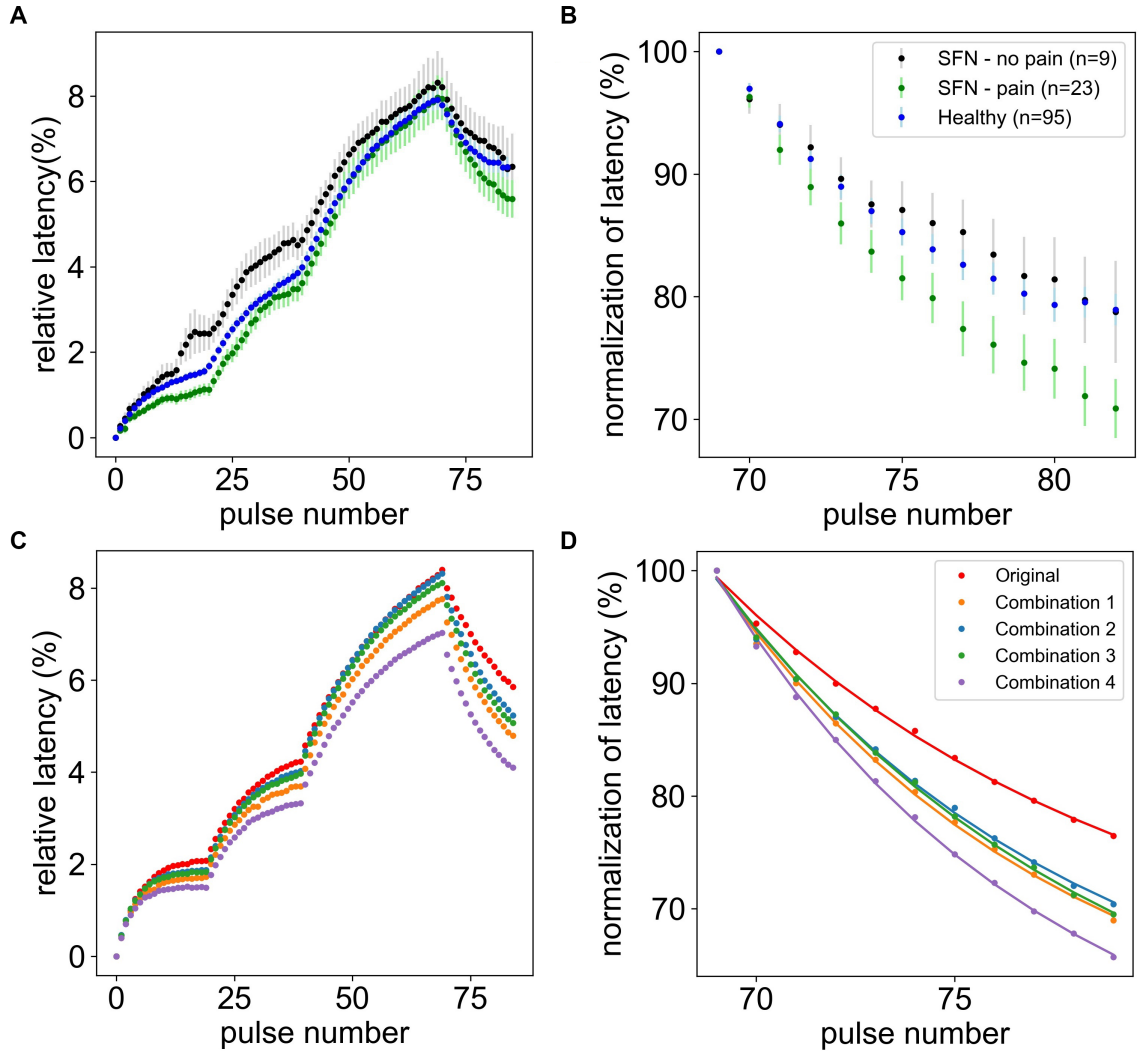
Comparison of microneurography data and simulated data. The upper panels show the mean and standard error of the mean of the relative latency of the full frequency protocol **(A)** and normalization of latency after stimulation **(B)** for healthy subjects (*n* = 95), pain (*n* = 23), and non-pain (*n* = 9) SFN patients. The lower panels show the best simulation results after the grid search compared to the original model (red) for the full frequency protocol **(C)** and normalization of latency after stimulation **(D)**. In combination 1 the channel is increased by 50% and the Na-K-pump is increased by 37.5%. In combination 2 the channel is increased by 37.5% and the channel h is reduced by 50%. In combination 3 the channel is increased by 50%, the Na-K-pump is reduced by 25% and the RMP is depolarized. In combination 4 the channel is increased by 50%, the channel h is increased by 50% and the RMP is depolarized.

In this study we are analyzing a large data set of CM and CMi-fibers of 19 SFN patients (substantially larger than those reported before (Kleggetveit et al., 2012)), providing a solid basis for computational modeling. We focused on ADS, relative latency, and normalization of latency in SFN patients with and without pain compared to healthy volunteers (Figures 4A,B). $\mathrm{AD}S_{\mathrm{total}}$ , the maximal latency observed for CMi-fibers, is similar for all groups (pain: 7.97% ± 0.51%, no pain: 8.32% ± 0.74%, healthy: 7.91% ± 0.24%, *p*-value = 0.811). In contrast, the normalization of latency is significantly quicker for the SFN patients with pain compared to those without or the healthy controls (pain: 70.89% ± 2.4%, no pain: 78.75% ± 4.17%, healthy: 78.93% ± 1.29%, *p*-value = 0.008). The post-hoc analysis showed that this difference is only significant between patients with pain and healthy subjects (*p*-value = 0.002), whereas the difference between patients with and without pain shows a tendency (*p*-value = 0.107). It has to be noted, that there were only a few no-pain patients (*n* = 9) because most SFN patients have ongoing pain in their extremities. Patients without pain and healthy subjects showed very similar behavior (*p*-value = 0.849). Note that the statements regarding significance for the post-hoc analysis consider a Bonferroni correction for multiple testing, leading to a significance level of α = 0.017 for comparison with the *p*-values.

### 3.3 Computational model: modulation of individual parameters

**Figure 5:**
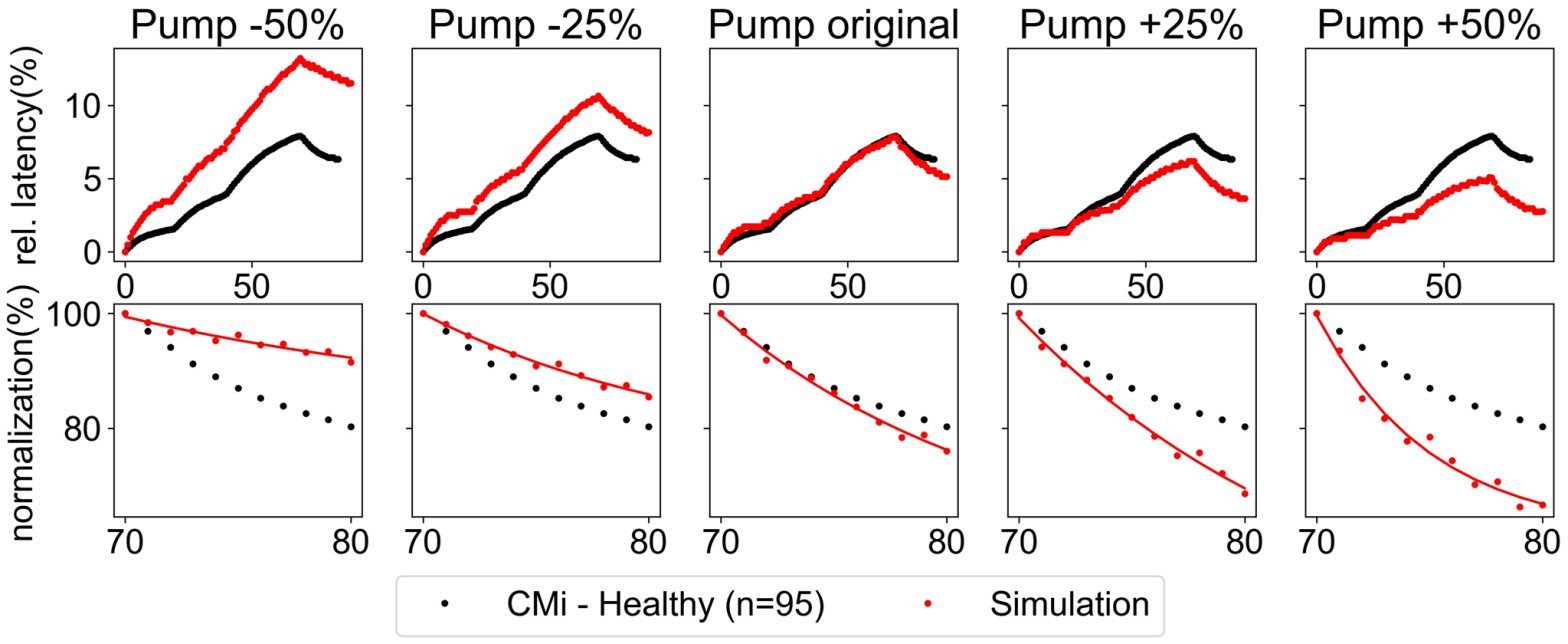
Variation of the Na-K-pump rate by ±25% and ±50% for the low protocol. Comparison of simulated data (red) and recorded data (black) of CMi-fibers from healthy subjects. The recorded data shows the mean and the standard error of the mean. Upper panel: relative latency for the full stimulation protocol. Lower panel: normalization of latency during the last ten pulses.

Now that we have identified significant differences in normalization of latency between patients with pain and healthy controls, we can use this data to optimize our model and investigate the possible underlying conductances for enhanced or decreased ADS or the speed of normalization. Using the computational model introduced in the methods, we explored how changes in the conductance of all ion channels, the Na-K-pump rate, and the resting membrane potential (RMP), affect the maximal $\mathrm{AD}S_{\mathrm{total}}$ and the total normalization of latency $rec_{\mathrm{total}}$ . The simulation results presented in Figure 5 show an example of this modulation for the Na-K-pump. It can be observed that, although an increasing Na-K-pump rate leads to a faster normalization of latency, simultaneously the ADS also changes substantially. Therefore, this variation did not improve the model.

#### 3.3.1 Na-K-pump, RMP, and $K_{dr}$ strongly affect ADS

**Figure 6:**
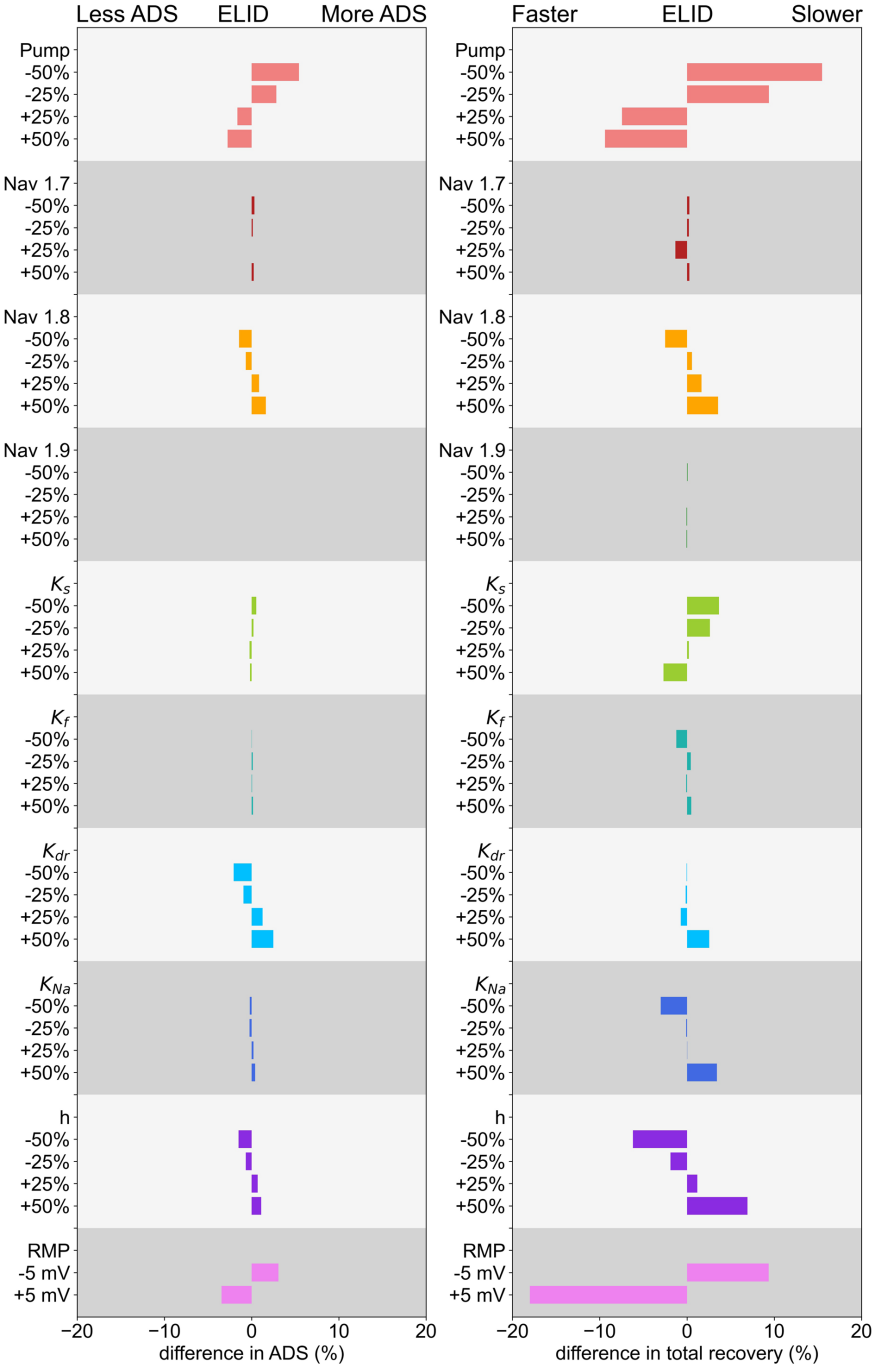
Effect of changing conductance of ion channels, Na-K-pump rate, and RMP of ADS on the left and normalization of latency on the right for a low-frequency protocol. The decrease/increase is shown in relation to the parameter value in the original model.

$\mathrm{AD}S_{\mathrm{total}}$ is mostly affected by changes in the Na-K-pump rate, the conductance of the potassium channel $K_{dr}$ and the RMP (Figure 6). An increase of the Na-K-pump rate as well as a depolarization of RMP leads to a reduced $\mathrm{AD}S_{\mathrm{total}}$ , while increasing conductance of $Na_{v}1.8\text{,}$ $K_{dr}$ and *h* has the opposite influence. The other channels only have a very slight impact on $\mathrm{AD}S_{\mathrm{total}}$ , while surprisingly $Na_{v}\;1.9$ , as a large persistent current, seems not to be relevant for the effect of ADS for this low frequency stimulation protocol.

#### 3.3.2 Normalization of latency is strongly influenced by the Na-K-pump, RMP and the hyperpolarization-activated current *h*

The simulation results and calculation of $rec_{\mathrm{total}}$ , shown in Figure 6, indicate that the Na-K-pump rate, the hyperpolarization-activated current *h*, and RMP have the most pronounced influence on the normalization of latency.

An increase in Na-K-pump rate or depolarization of RMP, as well as a decrease in the conductance of *h*, can lead to a faster normalization. But these mechanisms reduce at the same time the $\mathrm{AD}S_{\mathrm{total}}$ , which in pain patients is not different in comparison to healthy subjects. Further, increasing the conductance of the potassium channel $K_{s}$ , as well as reducing the conductance of the hyperpolarization activated current h , the potassium channel $K_{Na}$ or the sodium channels $Na_{v}\;1.7$ or $Na_{v}\;1.8$ also lead to a faster normalization, but to a smaller extent. Interestingly, different subtypes of one channel family, e.g., potassium channels, can have opposing effects on normalization. For example, a reduction of $K_{s}$ conduction leads to a slower normalization, while a reduction of $K_{Na}$ conduction results in a faster normalization. Thus, a very detailed analysis is important to understand C-fiber function.

#### 3.3.3 Adding the effects of individual mechanisms

Since no single mechanism can recreate all observed differences between SFN patients with pain and healthy subjects, we assessed different combinations of multiple mechanisms. Hence, we combined all possible variations of conductance levels, Na-K-pump rate, and RMP, which led to a total of 11,648 possible combinations. To run the model for all combinations is too computationally demanding, therefore for each combination we calculated the combined ADS and recovery from the previously calculated values $\mathrm{AD}S_{\mathrm{total}}$ and $rec_{\mathrm{total}}$ of the individual variations (Figure 6). Among these combinations were 18 that were able to reproduce the effect of unchanged ADS and faster normalization of latency. There was only one combination, where only two mechanisms were involved ( $\mathrm{Pump}+25\%,K_{dr}+25\%$ ) and 17 combinations where three mechanisms were involved. Eight of those 17 combinations consisted of Pump+25% and $K_{dr}+25\%$ and one other ion channel ( $Na_{v}\;1.7,Na_{v}1.9,K_{f},K_{Na}$ ) that had a negligible effect on the outcome. Therefore, we concluded that the main effect in these cases could be attributed to the Na-K-pump and $K_{dr}$ and thus summarize these combinations in the combination $\mathrm{Pump}+25\%,K_{dr}+25\%$ . The remaining combinations can be found in Table 2.

**Table 2:** Channel combinations, where the ADS is similar to the original model, while the normalization of latency is faster.

No.	Channel combinations	ADS difference to original model in % (absolute value)	Normalization difference to original model in %
1	$K_{f}-50\%,K_{dr}+25\%,h-50\%$	0.42	−10.21
2	$Na_{v}1.7+25\%,K_{dr}+50\%,h-50\%$	0.45	−6.79
3	Pump−50%,h−50%,RMP+5mV	0.47	−0.05
4	$\mathrm{Pump}+25\%,K_{dr}+25\%$	0.60	−3.81
5	$\mathrm{Pump}-25\%,K_{dr}+25\%,RMP+5\mathrm{mV}$	0.61	−6.65
6	$K_{dr}+50\%,h+50\%,RMP+5\mathrm{mV}$	0.88	−12.90
7	$\mathrm{Pump}+50\%,Na_{v}1.7+25\%,K_{dr}+50\%$	1.46	−9.23
8	$\mathrm{Pump}-25\%,Na_{v}1.7-50\%,RMP+5\mathrm{mV}$	1.60	−8.23
9	$\mathrm{Pump}-25\%,K_{Na}+25\%,RMP+5\mathrm{mV}$	1.70	−6.62
10	$\mathrm{Pump}-25\%,Na_{v}1.7+50\%,RMP+5\mathrm{mV}$	1.81	−7.11
	Experimental Data in Pain Patients (mean)	0.33	−8.8

Values for the difference in ADS and normalization of latency of channel combinations in comparison to the original model in ascending order for ADS. The goal is to have a low difference in ADS (<1%) and a faster normalization (in the range of −8.8% ± 4.5%). The fields in grey contain the combinations with the best fit.

Since the mechanisms influence each other, e.g., sodium influx influences the Na-K-pump rate, the identified combinations only give a rough indication of the optimal results. Therefore, the model was run with these 10 candidates of parameter sets and the result was compared to the original model (Figure A2 in Supplements). It can be observed that for some combinations the influence of the mechanisms on each other worsened the result for ADS and normalization of latency, i.e., some combinations have less ADS than the original, while others do not have a faster recovery. In Table 2 the exact numbers for the difference in ADS and normalization of latency compared to the original model are shown.

To find the best parameter sets, we need to look for those which have the lowest difference in ADS while simultaneously having a large difference in the normalization of latency. Therefore, the optimal parameter sets are numbers 1, 2, 5, and 6, marked in grey in Table 2.

### 3.4 Simultaneous modulation of multiple parameters within the computational model

To comprehend the interplay between ion channels, Na-K-pump, and RMP in greater depth, a grid search was performed for each of the four best combinations of mechanisms determined in the previous section. This approach additionally reveals mechanisms that have a negligible effect on ADS and latency normalization and identifies alternative parameter values for improved results. When visually inspecting the spike shapes of each resulting model, we found that seven models could not reproduce the original spike shape appropriately, see Figure A7, and were therefore excluded from further analysis. Further, it was confirmed that all gating variables reached steady state before the stimulation protocol starts.

#### 3.4.1 The potassium channel $K_{f}$ has low influence on ADS and normalization of latency

**Figure 7:**
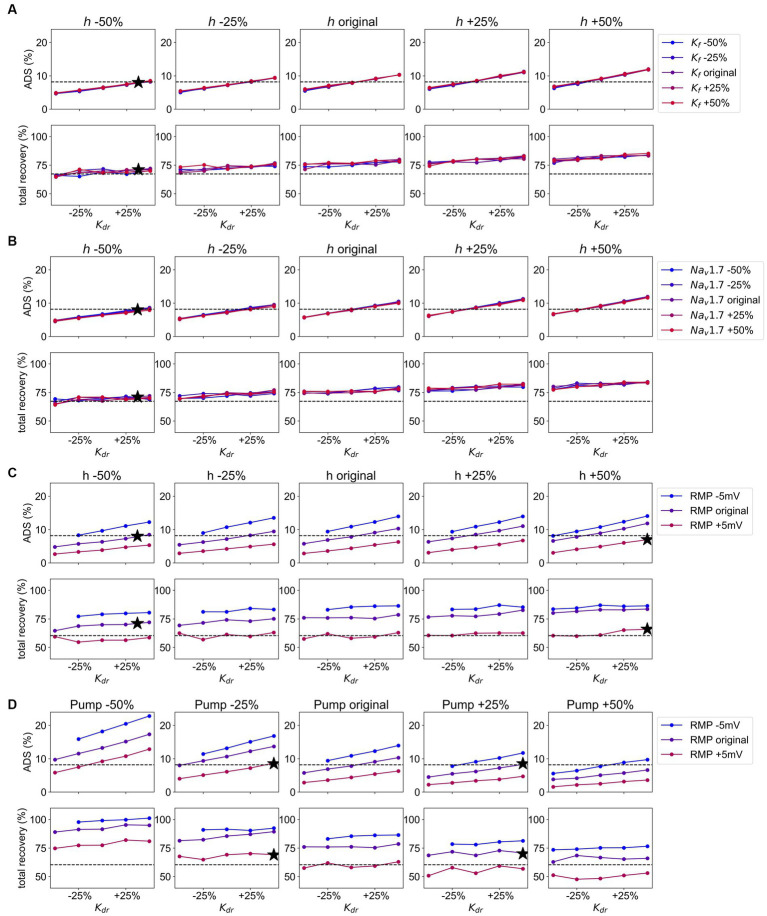
**(A)** Grid Search for the channels $K_{dr}$ , h , and $K_{f}\text{.}$ Values for $K_{dr}$ are given on the x-axis, from the left panel to the right conduction of h is increasing, and the increasing values for $K_{f}$ conduction are given in different colors from blue (decreased) to red (increased). The dotted line gives the value for ADS and normalization of latency, respectively as observed in pain patients. The black stars represent the optimal combination most similar to the values of ADS and normalization of latency in pain patients. Upper panel: the values for ADS for the model output with given parameter combinations. Lower panel: the values for normalization of latency for the model output with given parameter combinations. **(B)** Grid Search for the channels $K_{dr}$ , h , and $Na_{v}\;1.7\text{.}$ Values for $K_{dr}$ are given on the x-axis, from the left panel to the right conduction of h is increasing, and the increasing conduction of $Na_{v}\;1.7$ is given in different colors from blue (decreased) to red (increased). The dotted line gives the value for ADS and normalization of latency, respectively as observed in pain patients. The black stars represent the optimal combination most similar to the values of ADS and normalization of latency in pain patients. Upper panel: the values for ADS for the model output with given parameter combinations. Lower panel: the values for normalization of latency for the model output with given parameter combinations. **(C)** Grid Search for the channels $K_{dr}$ , h , and the RMP. Values for $K_{dr}$ are given on the x-axis, from the left panel to the right conduction of h is increasing, and the values for RMP are given different colors from blue (hyperpolarized) to red (depolarized). The dotted line gives the value for ADS and normalization of latency, respectively as observed in pain patients. The black stars represent the optimal combination most similar to the values of ADS and normalization of latency in pain patients. Upper panel: the values for ADS for the model output with given parameter combinations. Lower panel: the values for normalization of latency for the model output with given parameter combinations. **(D)** Grid Search for the channel $K_{dr}$ , the Na-K-pump, and the RMP. Values for $K_{dr}$ are given on the x-axis, from the left panel to the right Na-K-pump rate is increasing, and the values for RMP are given in different colors from blue (hyperpolarized) to red (depolarized). The dotted line gives the value for ADS and normalization of latency, respectively as observed in pain patients. The black stars represent the optimal combination most similar to the values of ADS and normalization of latency in pain patients. Upper panel: the values for ADS for the model output with given parameter combinations. Lower panel: the values for normalization of latency for the model output with given parameter combinations.

**Figure 8:**
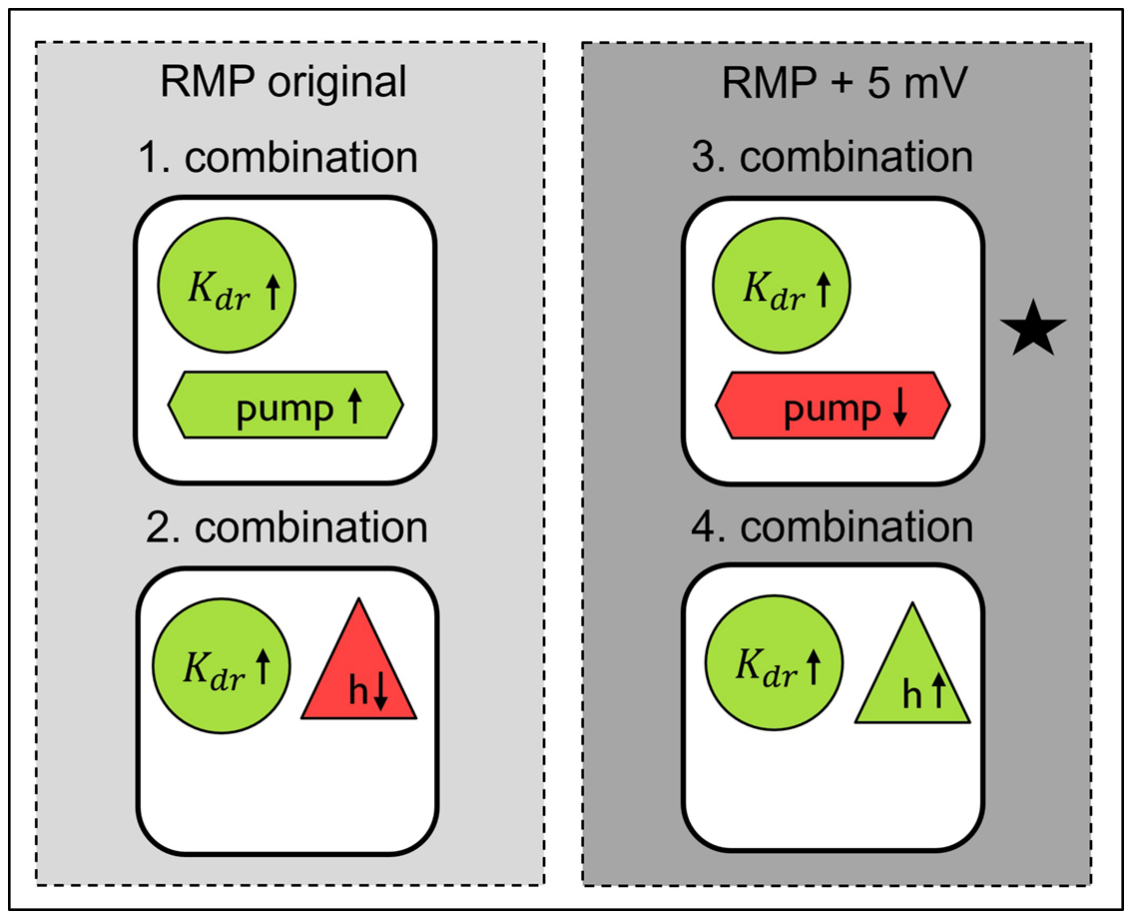
Best parameter combinations in computational model to simulate the changes observed in SFN patients with pain in comparison to healthy individuals and SFN patients without pain updated after grid search for original and depolarized RMP. Green indicates an increase in conductance or activity, while red indicates a decrease. The star indicates the combination which fits best to the pathological conditions previously observed in experimental work on neuropathy in human and in animal.

The results of the grid search for the ion channels $K_{dr}$ , h , and $K_{f}$ are depicted in Figure 7A. The colored lines corresponding to variations in the conductance of $K_{f}$ exhibit a minimal difference in terms of ADS and normalization of latency. Therefore, the effect of $K_{f}$ is low and its conductance can be maintained at its original value. Further, h and $K_{dr}$ influence each other, i.e., an increase in conductance of h results in a steeper line, indicating that variations in $K_{dr}$ conductance have a greater effect on ADS for higher conductance of h . Conversely, variations in conductance of h also have a larger effect for higher conductance of $K_{dr}$ (see Figure A3 in Supplements, here the lines are more dispersed for higher values of $K_{dr}$ conductance). However, no clear interaction between $K_{dr}$ and h is evident in the normalization of latency, see Figure 7A. The dashed line in the figures represents the optimal values for ADS and normalization of latency to match the behavior observed in SFN patients with pain (Kleggetveit et al., 2012), which is characterized by an increase in ADS by 0.33% and reduction in normalization by 8.8%. Therefore, in an optimal parameter set ADS and latency normalization should be simultaneously close to the dashed line, which is achieved by reducing the conduction of h by 50% and increasing the conduction of $K_{dr}$ by 37.5% (combination 2 in Figures 4, 8). Note that the optimal value for $K_{dr}$ is in between the calculated values. Since the behavior of the simulated fiber for ADS and normalization of latency is in this case almost linear, we can still conclude that this value would be optimal for $K_{dr}$ .

#### 3.4.2 The sodium channel $Na_{v}\;1.7$ does not influence ADS and normalization of latency

The findings of the grid search for the channels $K_{dr}$ , h , and $Na_{v}\;1.7$ are illustrated in Figure 7B. Similar to the previous grid search, it is visible that $Na_{v}\;1.7$ conduction has no effect on ADS or latency normalization and its variations can be disregarded. This leads to the same optimal parameter combination as in the previous section, where conduction of h is reduced by 50% and $K_{dr}$ conductance is increased by 37.5% (combination 2 in Figures 4, 8), and the prior observations about the influence of $K_{dr}$ and h on each other hold true in this scenario as well.

#### 3.4.3 The ion channels $K_{dr}$ and h and RMP strongly influence ADS and latency normalization

The results of the grid search for the channels $K_{dr}$ , h , and the RMP are depicted in Figure 7C. Both channels and the RMP have a noticeable effect on ADS and latency normalization unlike in the previous grid searches. For increased conductance of h variations in $K_{dr}$ conductance affect the ADS more, which can be observed through increasingly steeper lines. Interestingly, variations in conductance of h only affect ADS and normalization when the RMP is maintained at its original value. On the other hand, variations in conductance of $K_{dr}$ have a slightly more pronounced effect on ADS, if the RMP is hyperpolarized, which can be best observed Figure A5 in Supplements, where the steepness of the lines increases for hyperpolarized RMP. Two options for achieving an unchanged ADS and increased normalization as observed in pain patients can be identified. The first option involves retaining the RMP at its initial value, increasing $K_{dr}$ conduction by 37.5% and simultaneously reducing h conduction by 50%, resulting in the same parameter set as previously identified in both prior grid searches (combination 2 in Figures 4^8^). The second option involves depolarizing the RMP by 5 mV, increasing h conduction by 50% and $K_{dr}$ conduction by 50% (combination 4 in Figures 4^8^). Note that depolarizing the RMP leads to an increase in h conduction instead of a decrease.

#### 3.4.4 The potassium channel $K_{dr}$ , the Na-K-pump and RMP influence ADS and latency normalization

The results of the grid search modulating the parameters $K_{dr}$ , the Na-K-pump and the RMP are depicted in Figure 7D. A reduction in Na-K-pump rate leads to an amplified effect of $K_{dr}$ conduction and RMP on ADS, as seen in Figure 7D as increased dispersion of the lines and steeper lines for lower Na-K-pump rate. However, this effect is not reflected in the normalization of latency, where the dispersion and steepness of the lines is similar for all values of the Na-K-pump rate. Further, an increase in $K_{dr}$ conduction results in a slightly larger influence of variations of the Na-K-pump rate on both the ADS and normalization as demonstrated in Figure A4 in the Supplements, where the dispersion of the lines is slightly larger. A hyperpolarization of RMP results in a heightened effect of both $K_{dr}$ conduction and the Na-K-pump rate on the ADS as depicted in Figure A5 of the Supplements. Based on this grid search, two options for parameter sets have been identified that achieve our desired effect of unchanged ADS and reduced normalization of latency. For a depolarized RMP the optimal approach is to increase $K_{dr}$ conduction by 50% and decrease the Na-K-pump rate by 25% (combination 3 in Figures 4, 8). For an unchanged RMP, a 37.5% increase of the Na-K-pump rate achieves the desired effect (combination 1 in Figures 4, 8), which can be best observed in Figure A5 in the Supplements. It needs to be noted that the optimal value for Na-K-pump is in between the calculated values, which can be accepted due to the linear behavior of the simulated fiber in this scenario.

#### 3.4.5 Summary of optimized parameter combinations

By conducting grid searches, we were able to optimize previously identified parameter values and identify mechanisms with low influence on ADS and normalization of latency, i.e., the conduction of the ion channels $K_{f}$ and $Na_{v}\;1.7$ . Additionally, we discovered a previously unknown parameter set (increased Na-K-pump rate by 37.5% and an increased $K_{dr}$ conduction by 50%). A schematic overview of the resulting parameter combinations is presented in Figure 8.

Executing the computational model with these parameter sets and a long axon of 10 cm, showed that the model performance had improved compared to previous results (Figures 4C,D). For three combinations ADS is near the original value, while simultaneously the normalization of latency is decreased sufficiently. For the last combination all parameter values were already optimal within the parameter space and could not be further improved. A figure showing the individual contributions of each ionic current during an action potential for the four final combinations can be reviewed in the Supplementary Figures (Figure A6).

## 4 Discussion

### 4.1 Model adaptations

In this study we employed a computational model to reproduce alterations in axonal propagation properties in C-fibers observed in small fiber neuropathy (SFN) patients with pain. We based our optimization on a large human data set derived from about 100 CM and CMi-fibers from SFN patients with and without pain and healthy controls. The computational model allows the observation and controlled manipulation of multiple parameters such as resting membrane potential (RMP), ion channel conductance and Na-K-pump rate. We were able to accurately replicate the observed changes and identified potential responsible mechanisms, shedding light on the underlying biological processes involved. For parameter optimization we applied a grid search method, where the model was executed 75 to 125 times per combination depending on the number of parameter changes. Therefore, the execution time of the model was crucial. We adjusted an existing nerve fiber model for C-fibers (Tigerholm et al., 2014) by reducing the length of the fiber from 10 cm to 1 cm and changing the morphology to a cylinder with uniform diameter and temperature. This led to a reduced computation time from over 1 h to just 7–10 min, which is crucial when considering the repetitive executions of 75 to 125 times in the grid search approach. We confirmed the fit of the model for CMi-fibers by comparing the result to microneurography data from healthy subjects and adjusted the diameter according to the best fit for the data. This validation with a large set of human data ensures a high quality of the computational model, which can be used to study potential ionic mechanisms underlying pain-related pathophysiological alterations in excitability.

### 4.2 Alterations in pain patients validated with previously unpublished data

In our previous publication with only a small patient number we found a more pronounced normalization of latency in pain patients in contrast to non-pain patients during lower stimulation frequency after a train of higher frequency stimulation (Kleggetveit et al., 2012). ADS can be seen as a self-inhibiting mechanism: more activity causes more ADS which is an indicator of hypoexcitability, because of accumulating sodium channel inactivation. Thus, a quicker normalization would indicate relative hyperexcitability for the amount of previous activity and enable the fiber to discharge quicker following an AP and potentially producing more action potentials. Since ongoing pain in the feet is a typical and common symptom of SFN, it is hard to find SFN patients without ongoing pain in the feet and thus also the new group of patients without pain is small (Rasmussen et al., 2004). We thus added a large number of CMi-fibers from healthy volunteers. We compared the total ADS and the normalization of latency of SFN patients with and without pain with healthy volunteers. All groups showed no significant difference to each other in total ADS as observed before (Kleggetveit et al., 2012). Even though the difference between pain and non-pain patients was not significant, we observed that the course of the curve for non-pain patients and healthy subjects was very similar. Therefore, the non-significance could also be caused by the small sample size (9 non-pain patients) and a resulting higher standard error. This has to be investigated in further studies with larger sample sizes before we can draw definite conclusions. However, the normalization of latency of pain patients was significantly faster than for healthy subjects. While the magnitude of the difference (8.04%) might seem small, it remains a valid observation when compared with other parameters obtained in microneurographic recordings. While our results are not identical with the findings of Kleggetveit et al., they reflect the general trend that SFN patients with pain seem to have a faster recovery. Our results offer an explanation for the hyperexcitability and spontaneous activity in mechanoinsensitive C-nociceptors that are associated with spontaneous pain in peripheral neuropathy (Ochoa et al., 2005; Kleggetveit et al., 2012).

### 4.3 Individual mechanisms with largest impact on ADS and normalization

Via our model of a human C-fiber axon we set out to explore possible molecular mechanisms accounting for quicker normalization of latency, combined with unchanged ADS, a feature of CMi-fibers we observed in the SFN patients suffering from pain. We first varied single parameters individually. We found that changes in RMP and in the Na-K-pump rate have the greatest influence on both, ADS and normalization of latency. A reduction in Na-K-pump rate leads to an increase in ADS and a slower normalization of latency, which is an effect that can also be observed in elderly subjects (Namer et al., 2009). A reduction in Na-K-pump conductance would lead to accumulation of intracellular sodium (Tigerholm et al., 2014), which would result in increased ADS (De Col et al., 2008). Also, the potassium channel $K_{dr}$ has a notable effect on ADS, i.e., a decrease in $K_{dr}$ conduction leads to less ADS. It was proposed that the reduction of $K_{dr}$ conduction could cause the membrane potential to repolarize slower after the peak of the action potential and therefore prevent recovery from slow inactivation of sodium channels (Petersson et al., 2014). This would lead to a reduction of sodium current in the subsequent action potential and ultimately less ADS (Petersson et al., 2014). Further, the hyperpolarization activated current *h*, which plays an important role during repetitive firing, largely affects the normalization. All variations of individual mechanisms have qualitatively the same effect on ADS as in Peterson et al., where a similar computational model was used with a different stimulation protocol to assess how variations in parameters affect the ADS (Petersson et al., 2014). The normalization of latency was not assessed there.

### 4.4 Combinations of multiple mechanisms reproducing stable ADS and faster normalization

In a nerve cell as well as in the computational model the conductance of different ion channels, the Na-K-pump rate and the RMP are all highly dependent on each other. Therefore, we investigated the interplay between these mechanisms and found several combinations which could replicate the effect of quicker normalization of latency as observed in SFN patients with pain. By adding the effects for ADS and normalization of latency for all possible combinations of variations of mechanisms, we discovered 18 candidates that could replicate the effect of faster normalization and stable ADS. We executed the model with these parameter combinations. We chose the best four combinations for further investigation and starting points in a grid search, since a grid search over all possible combinations would be too time consuming. This allowed us to optimize the parameter sets by finding the optimal value for each parameter and identifying parameters with low influence on the result, such as the conductance of the potassium channel $K_{f}$ and the sodium channel $Na_{v}\;1.7$ . Additionally, via this grid search approach we discovered a previously unknown combination consisting of an increased Na-K-pump rate by 37.5% and an increased $K_{dr}$ conduction by 50%. The grid search revealed further that the Na-K-pump and the hyperpolarization activated channel *h* have the opposite effect of each other, i.e., a reduction of the Na-K-pump rate leads to the same effect as an increase in conduction of h and vice versa. This is not surprising since the Na-K-pump drives sodium out of the cell and potassium into the cell, while h does the opposite. Nevertheless, the kinetics are vastly different, and activation of *Ih* occurs only at hyperpolarized potentials. The results suggest that *Ih* is active at RMP, especially, as we observed the opposite effect also on the RMP, which is an important mediator of the ADS and normalization. An increased Na-K-pump rate leads to a more hyperpolarized RMP, while an increase in conduction of h leads to a more depolarized RMP (Zhang et al., 2017). We found that the Na-K-pump and h are responsible for the faster normalization, but simultaneously decrease ADS. Therefore, the channel $K_{dr}$ is needed to increase the ADS and bring it back to its original value. The combination of the mechanisms then leads to a stable ADS, while having a faster normalization. Interestingly, a depolarization of the RMP leads to the need for the Na-K-pump rate and the conduction of h to be adjusted in different directions to achieve the same effect, i.e., for a depolarized RMP the Na-K-pump rate needs to be reduced, while for the original RMP the Na-K-pump rate needs to be increased. The depolarization of the RMP leads to a much faster normalization of latency and less ADS, which is balanced out by increasing the conduction of h or decreasing the Na-K-pump rate.

Further, we elucidated via the grid search the interplay between different mechanisms. Especially, modulation of the Na-K-pump rate influenced the impact of other mechanisms on the result. A reduction of Na-K-pump rate causes the RMP and $K_{dr}$ conduction to have a larger impact on the result, i.e., smaller changes in the RMP and $K_{dr}$ conduction create larger differences in ADS. This shows that the interplay between different mechanisms is complex and hard to assess experimentally and therefore an investigation with computational models is helpful.

### 4.5 Physiological and clinical relevance

We set out to examine the influence of voltage gated sodium channels on the faster normalization of latency after a train of action potentials with a higher frequency as the importance of sodium channels for hyperexcitability of nociceptors in painful neuropathies has been shown in many studies (Cox et al., 2006; Fertleman et al., 2006; Goldberg et al., 2007; Huang et al., 2013; Brouwer et al., 2014). Additionally ADS is strongly influenced by sodium channel inactivation and intracellular sodium accumulation and associated with excitability of the neuron (De Col et al., 2008; Tigerholm et al., 2014). The slower a neuron conducts due to previous activity the less excitable it gets due to sodium channel inactivation and intracellular sodium accumulation. Therefore, ADS can be seen as self-inhibiting mechanism which protects from dangerous hyperactivity. Thus, when after certain activity the neuron returns quicker to its original conduction, the excitability necessary for producing action potentials again is reached quicker and higher discharge rates are possible. Interestingly, normalization turned out in our model to be modulated by different mechanisms than ADS, namely RMP, Na-K-pump, *h* and $K_{dr}$ . Although this is still under debate, there are indications that the RMP could be depolarized in diabetic neuropathy (Krishnan and Kiernan, 2005). Additionally, patients with diabetic neuropathy show a reduced Na-K-pump function (Krishnan et al., 2008). Some peripheral neuropathies can be caused by mitochondrial disorders (Pareyson et al., 2013; Lee et al., 2020), which lead to a loss of energy in the cell and this could cause a reduced Na-K-pump rate. Taking those previous findings into account, the combination in Figure 8, which is marked with a black star, is the most likely to be present in patients. In our model in this specific combination of mechanisms the RMP is depolarized, the Na-K-pump rate is decreased and the channel conductance $K_{dr}$ is increased. The reduced function of some $K_{dr}$ channels leads to less mechanical and heat pain (Tsantoulas and McMahon, 2014). Here, we speculate that the opposite might also be true, i.e., an increase in conduction of $K_{dr}$ might be associated to more pain via the above discussed mechanisms. Our model showed that in other combinations of mechanisms the hyperpolarization activated current *h* played a significant role. In rodent models blocking this channel leads to reduced discharge frequencies and reduced pain behavior (Chaplan et al., 2003; Jiang et al., 2008; Bernal and Roza, 2018), as well as decreases mechanical hyperalgesia (Luo et al., 2007; Young et al., 2014; Tsantoulas et al., 2017). Conversely, there are also studies which suggest the opposite, namely that blocking of *h* channels increases neuronal firing (Doan et al., 2004), which might lead to more pain. In the latter study the RMP was hyperpolarized, which could be an indication that the differences in these studies could be explained by different RMPs and point out the important role RMP plays. In Figure 8 we can observe that changes in the RMP lead to the need to adjust conduction of *h* in the opposite direction to achieve the same effect, i.e., for a depolarized RMP the conductance of *h* needs to be increased while for the original RMP the conductance of *h* needs to be decreased.

RMP alterations seem crucial to determine which mechanisms in the end are responsible for hyperexcitability in patient’s nociceptors so that an individual variability in mechanisms leading to nociceptor hyperexcitability exists which calls for stratified or even personalized medicine in the treatment of neuropathic ongoing pain. It may also be possible that the mechanisms may not always be identical, and that several changes lead to similar results. This is underlined by the identification of several sets of parameters which are able to induce similar outcomes.

Mechanistically, a moderately depolarized membrane potential can be associated with hyperexcitability of nociceptors: A reduced rate of the Na-K-pump, as identified here in our simulations, supports depolarization. Depolarization leads to an increased fraction of inactivated sodium channels resulting in hypoexcitability, while at the same time it moves membrane voltage closer to the Na_v_ activation threshold, supporting hyperexcitability. However, it is unclear which mechanism predominates in determining excitability. Increased conductance of $K_{dr}$ channels might lead to quicker and/or stronger repolarization following an action potential resulting in a higher fraction of available sodium channels supporting excitability. Thus, the identified mechanisms can contribute to hyperexcitability, and offer a potential peripheral mechanism of ongoing pain in some SFN patients with neuropathic pain.

Although significant differences in morphology and ion channel subtype composition are found in central nervous system (CNS) neurons and other experimental model neurons like in invertebrates, they show similar activity dependent changes of the nerve fiber conduction as unmyelinated peripheral primary nociceptive afferents (Zhang et al., 2017; Roth and Hu, 2020). Even if displayed in each study differently, ADS can be found in axons of CNS neurons and even in crustacean stomatogastric neurons (Zhang et al., 2017). In those publications, *h*, resting membrane potential and Na-K-pump is assumed to have major influence on activity-dependent modulation of axonal action potential conduction (Zhang et al., 2017; Roth and Hu, 2020). Thus, the contributions of Na-K-pump and *h* seem to be general peripheral and central axonal and evolutionary conserved mechanisms regulating the short-term memory of axons. Additionally, it was demonstrated in rodents that *h* and Na-K-pump both play an important role in the regulation of the resting membrane potential (Kim et al., 2007; Roth and Hu, 2020). Specifically, *h* depolarizes the RMP, while Na-K-pump hyperpolarizes it, when fibers are stimulated by high-frequency trains of spikes (Kim et al., 2007; Roth and Hu, 2020). It is still unclear if this also accounts for low frequency stimulation of C-fibers activity-dependent slowing as shown in the current study.

### 4.6 Limitations

The computational modeling of nerve cells is a valuable tool for studying neuronal function and behavior. The interplay of ion channels and other mechanisms can be evaluated without further inventions in humans. However, a computational model will always differ from a real nerve fiber in some respects. To be able to perform the necessary computations for our study, we had to reduce the complexity of the model. This was achieved by simplifying the morphology of the model, i.e., reduce the length of the fiber and have a uniform diameter and temperature across the fiber. In a real nerve fiber, diameter and temperature vary along its length, with a thinner and cooler peripheral part. Since we assessed only a very short fiber this change could be neglected.

In the newly assessed data, the difference between SFN patients with and without pain could only be shown indirectly by comparing both groups with healthy subjects, due to a small sample size of patients without pain, since most patients with SFN have pain in the extremities. Nevertheless, the difference between healthy subjects and SFN patients with pain is in a similar range. The normalization of latency was nearly identical in healthy volunteers and patients without pain suggesting that a larger sample size of patients without pain might have produced a significant difference between patients with and without pain in the feet.

### 4.7 Future study considerations

In the current paper, we assessed the effects of regular stimulation in different frequencies over the time course of minutes on following latency normalization. In spontaneous activity and after chemical stimulation of nerve fibers a bursting behavior is often observed, i.e., the fiber exhibits a pattern of activity characterized by a series of action potentials that occur in rapid succession, followed by a brief period of inactivity. Since different mechanisms could modulate the effect of these burst, exploring this phenomenon further could be a valuable direction for future research. For this purpose, we propose to use a protocol mimicking these irregular discharges in small trains of action potentials. This protocol can provide valuable insights into the short-term effects on normalization and could be used in future computational studies to investigate the mechanisms involved. Since normalization is thought to play a key role in chronic pain conditions, using this protocol in microneurography experiments in healthy volunteers and SFN patients with and without pain could further deepen the understanding in differences between these groups. Overall, investigating these mechanisms can improve our understanding of the pathophysiology of pain and lead to the development of new therapeutic strategies.

## 5 Conclusion

Our study assesses the complex interplay between various ion channels, sodium potassium pump and resting membrane potential (RMP) modulating the normalization of latency. Our findings confirm that the normalization of latency is an important marker identifying pathological function of CMi-fibers of SFN patients with and without pain and healthy subjects, even when ADS is unchanged. In the past the importance of sodium channels in neuropathic pain disorders was often highlighted and they are undoubtedly a human validated pain target, as humans lacking functional Na_v_1.7 are pain free. In the present study, we have indications that RMP, the Na-K-pump, the potassium channel $K_{dr}$ and the hyperpolarization-activated current *h* are important modulators of normalization of latency and contribute to the pathologically modified CMi-fibers of SFN patients with ongoing pain. Moreover, we demonstrated that changes in the RMP have a major influence on other mechanisms so that opposite changes in conductance, result in similar effects on CMi-fiber function. Overall, these findings offer new avenues for targeted therapeutic interventions and highlight the potential of computational models in medical research.

## Data availability statement

The datasets presented in this article are not readily available because of ethical and privacy restrictions. Requests to access the datasets should be directed to BN, bnamer@ukaachen.de.

## Ethics statement

The studies involving humans were approved by Ethics committees from Aachen (EK 141/19) and Erlangen (4361). The studies were conducted in accordance with the local legislation and institutional requirements. The participants provided their written informed consent to participate in this study.

## Author contributions

AM: Formal analysis, Software, Visualization, Writing – original draft, Writing – review & editing. EK: Methodology, Supervision, Writing – review & editing. MD: Data curation, Writing – review & editing. PS: Methodology, Supervision, Writing – review & editing. AL: Methodology, Validation, Writing – review & editing. JT: Conceptualization, Methodology, Supervision, Writing – review & editing. BN: Conceptualization, Data curation, Methodology, Resources, Supervision, Writing – review & editing.

## Supplementary material

SUPPLEMENTARY FIGURE A1 Performed workflow to find optimal parameter combinations, which reproduce the changed normalization of latency for SFN patients with pain in comparison to non-pain patients and healthy individuals. The grey boxes contain the performed computational steps and the additional text explains the rationale behind each step and the results obtained from it.

SUPPLEMENTARY FIGURE A2 ADS and relative latency for different combinations of ion channels, pump and resting membrane potential (RMP): in the left panel the relative latency for the full protocol is shown for different combinations of mechanisms compared to the original (black); in the right panel the relative latency for the last 10 pulses of the protocol is shown. The latency is normalized to the highest value of the ADS, so that all models start at 100%.

SUPPLEMENTARY FIGURE A3 Grid Search for the channels *K**_dr_*, *h*, and *K**_f_*. Values for *K**_f_* are given on the x-axis, from the left panel to the right *K**_dr_* is increasing, and the increasing values for *h* are given in different colors from blue to red. The dotted line gives the desired value for ADS and normalization of latency, respectively. Upper panel: the values for ADS for the model output with given parameter combinations. Lower panel: the values for normalization of latency for the model output with given parameter combinations.

SUPPLEMENTARY FIGURE A4 Grid Search for the channel *K**_dr_*, the Na-K-pump and RMP. Values for RMP are given on the x-axis, from the left panel to the right *K**_dr_* is increasing, and the increasing values for the Na-K-pump are given in different colors from blue to red. The dotted line gives the desired value for ADS and normalization of latency, respectively. Upper panel: the values for ADS for the model output with given parameter combinations. Lower panel: the values for normalization of latency for the model output with given parameter combinations.

SUPPLEMENTARY FIGURE A5 Grid Search for the channel *K**_dr_*, the Na-K-pump and RMP. Values for *K**_dr_* are given on the x-axis, from the left panel to the right RMP is increasing, and the increasing values for the Na-K-pump are given in different colors from blue to red. The dotted line gives the desired value for ADS and normalization of latency, respectively. Upper panel: the values for ADS for the model output with given parameter combinations. Lower panel: the values for normalization of latency for the model output with given parameter combinations.

SUPPLEMENTARY FIGURE A6 The ionic currents during an action potential for the four final combinations of the grid search. The panels on the left show all ionic currents, the panels on the right only the small ionic currents.

SUPPLEMENTARY FIGURE A7 Spike shape of all performed model evaluations. For Grid Search A (for the channels *K**_dr_*, *h*, and *K**_f_*) and Grid Search B (for the channels *K**_dr_*, *h*, and *Na**_v_* 1.7) all spike shapes resemble qualitatively the original model, while for Grid Search C (for the channels *K**_dr_*, *h*, and the *RMP*) and Grid Search D (for the channel *K**_dr_*, the Na-K-pump, and the *RMP*) some spikes show strange behavior and were excluded from the further analysis.
