# Supplementary figures and images for: A modelling study to dissect the potential role of voltage-gated ion channels in activity-dependent conduction velocity changes as identified in small fiber neuropathy patients
Source: Front Comput Neurosci. 2023 Dec 14;17:1265958. doi: 10.3389/fncom.2023.1265958 (PMC10752960; doi:10.3389/fncom.2023.1265958)

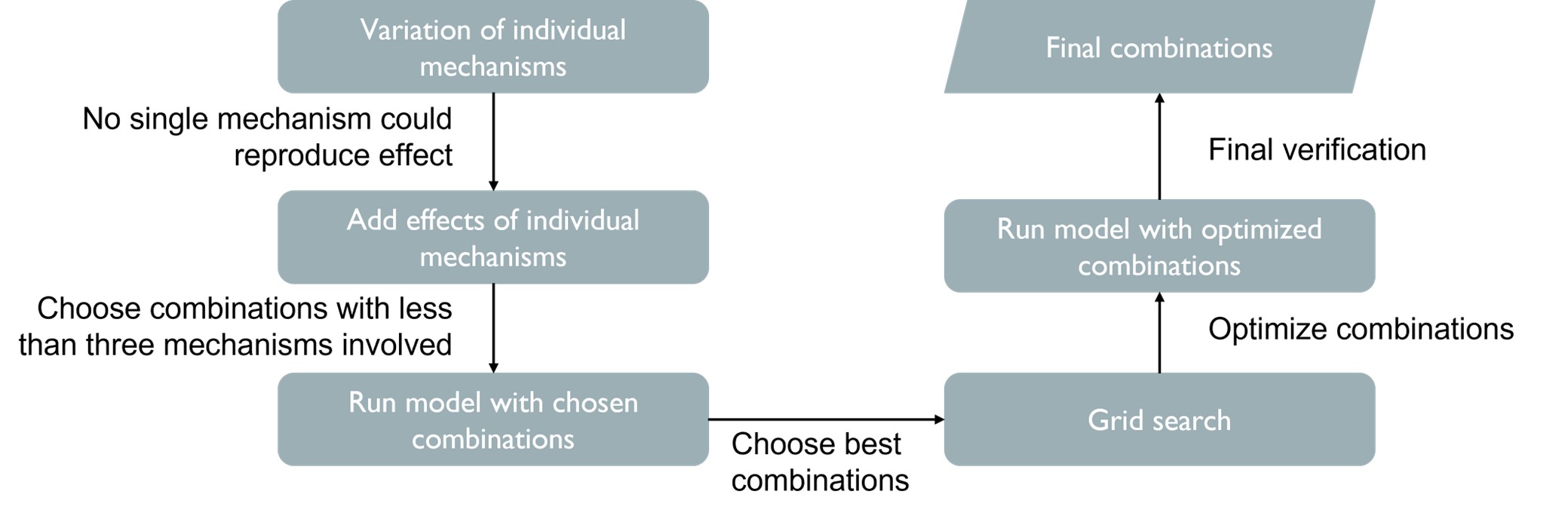

Supplement: SUPPLEMENTARY FIGURE A1 — Performed workflow to find optimal parameter combinations, which reproduce the changed normalization of latency for SFN patients with pain in comparison to non-pain patients and healthy individuals. The grey boxes contain the performed computational steps and the additional text explains the rationale behind each step and the results obtained from it. [file Image_1.JPEG]

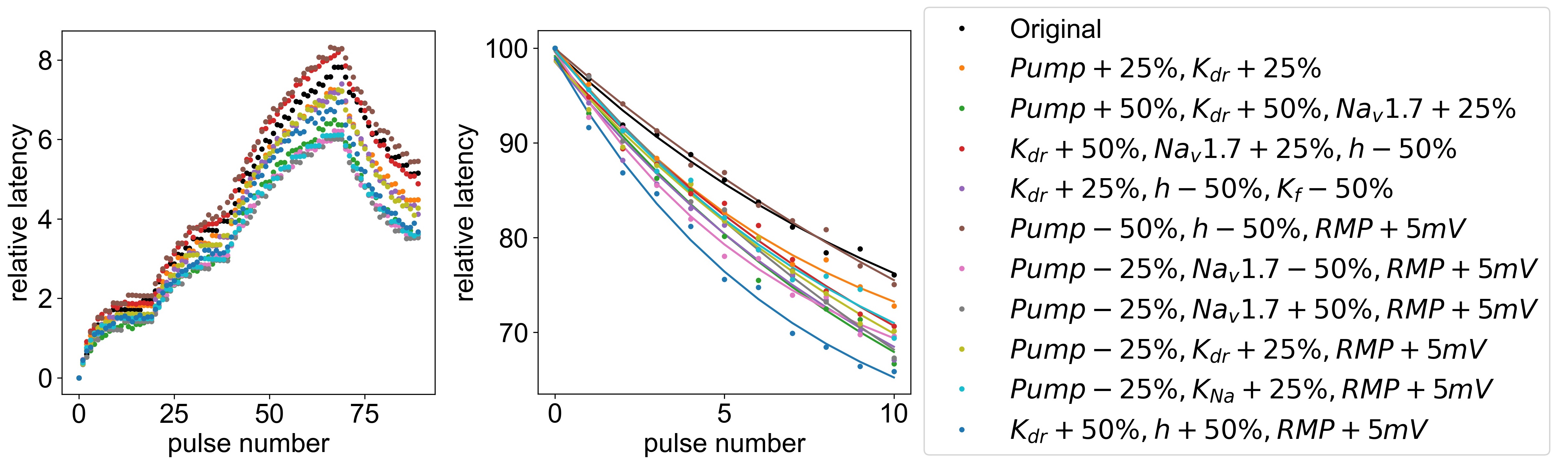

Supplement: SUPPLEMENTARY FIGURE A2 — ADS and relative latency for different combinations of ion channels, pump and resting membrane potential (RMP): in the left panel the relative latency for the full protocol is shown for different combinations of mechanisms compared to the original (black); in the right panel the relative latency for the last 10 pulses of the protocol is shown. The latency is normalized to the highest value of the ADS, so that all models start at 100%. [file Image_2.JPEG]

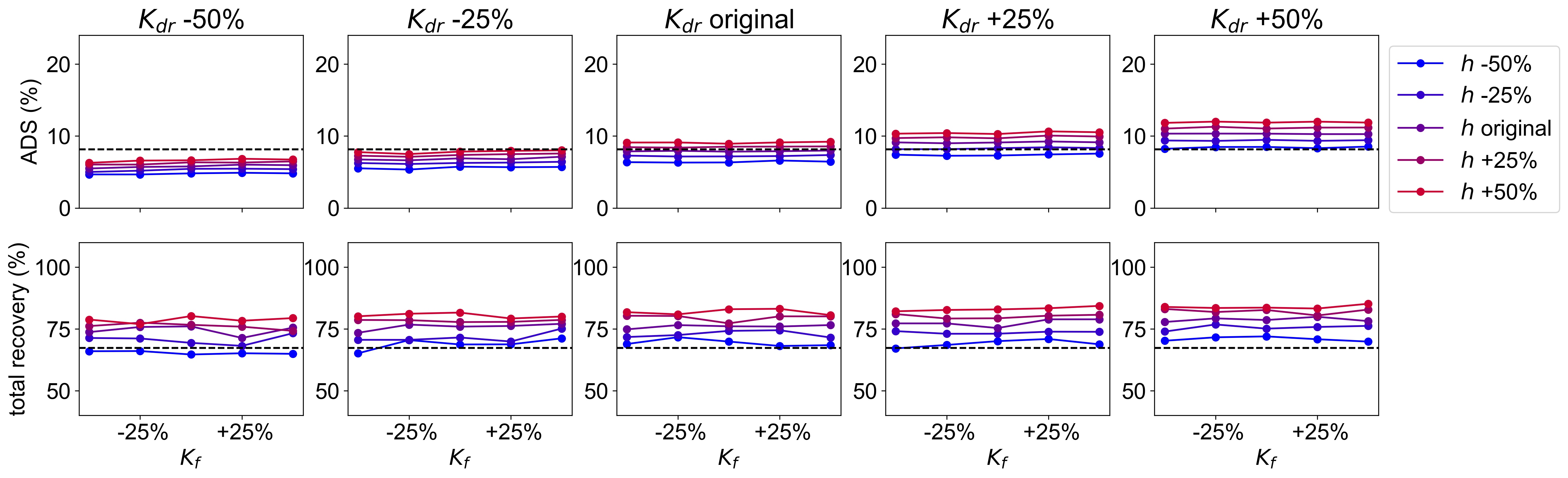

Supplement: SUPPLEMENTARY FIGURE A3 — Grid Search for the channels K dr , h, and K f . Values for K f are given on the x-axis, from the left panel to the right K dr is increasing, and the increasing values for h are given in different colors from blue to red. The dotted line gives the desired value for ADS and normalization of latency, respectively. Upper panel: the values for ADS for the model output with given parameter combinations. Lower panel: the values for normalization of latency for the model output with given parameter combinations. [file Image_3.JPEG]

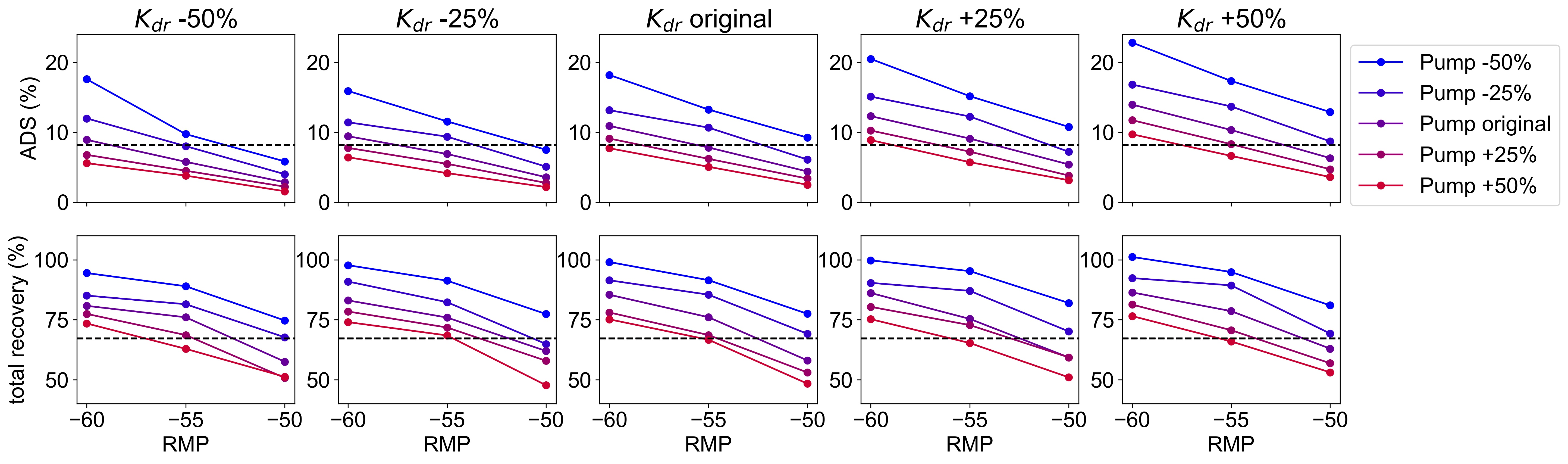

Supplement: SUPPLEMENTARY FIGURE A4 — Grid Search for the channel K dr , the Na-K-pump and RMP. Values for RMP are given on the x-axis, from the left panel to the right K dr is increasing, and the increasing values for the Na-K-pump are given in different colors from blue to red. The dotted line gives the desired value for ADS and normalization of latency, respectively. Upper panel: the values for ADS for the model output with given parameter combinations. Lower panel: the values for normalization of latency for the model output with given parameter combinations. [file Image_4.JPEG]

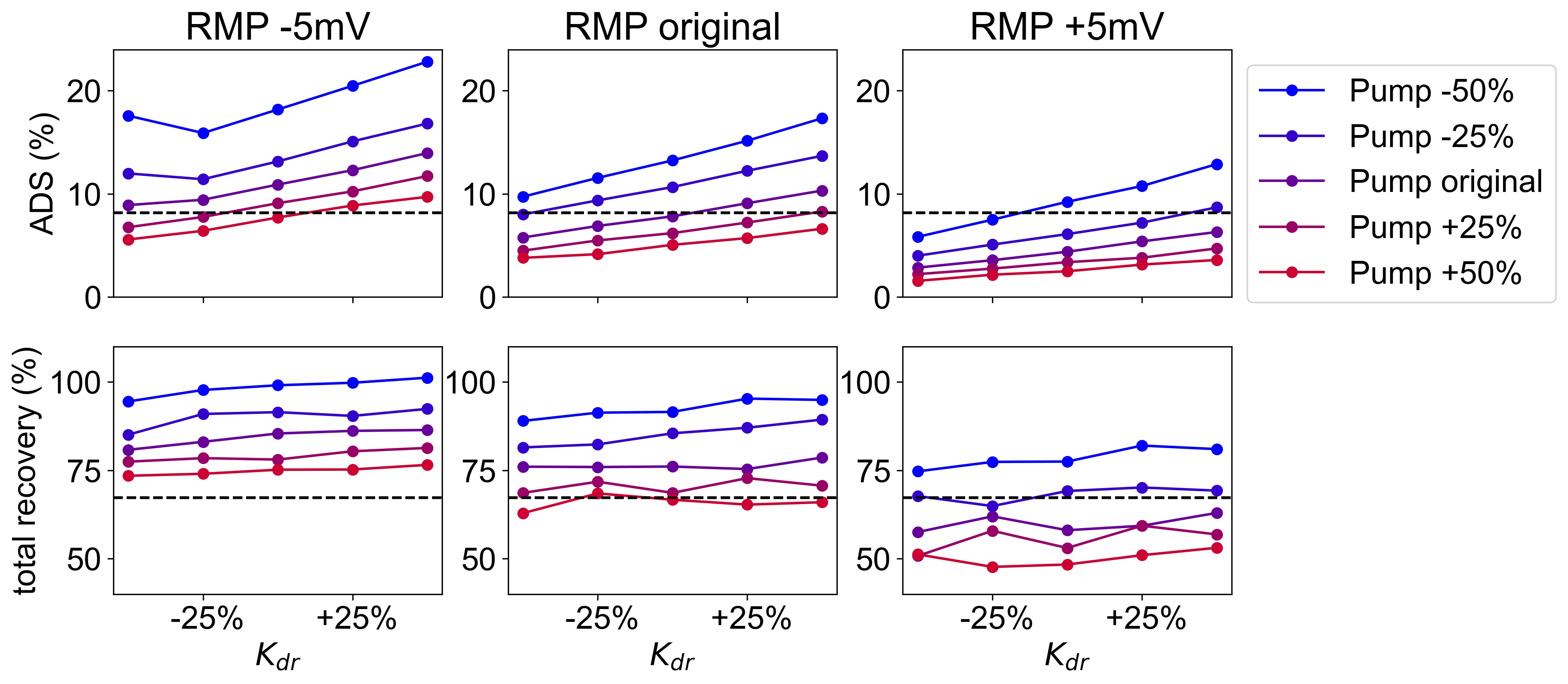

Supplement: SUPPLEMENTARY FIGURE A5 — Grid Search for the channel K dr , the Na-K-pump and RMP. Values for K dr are given on the x-axis, from the left panel to the right RMP is increasing, and the increasing values for the Na-K-pump are given in different colors from blue to red. The dotted line gives the desired value for ADS and normalization of latency, respectively. Upper panel: the values for ADS for the model output with given parameter combinations. Lower panel: the values for normalization of latency for the model output with given parameter combinations. [file Image_5.JPEG]

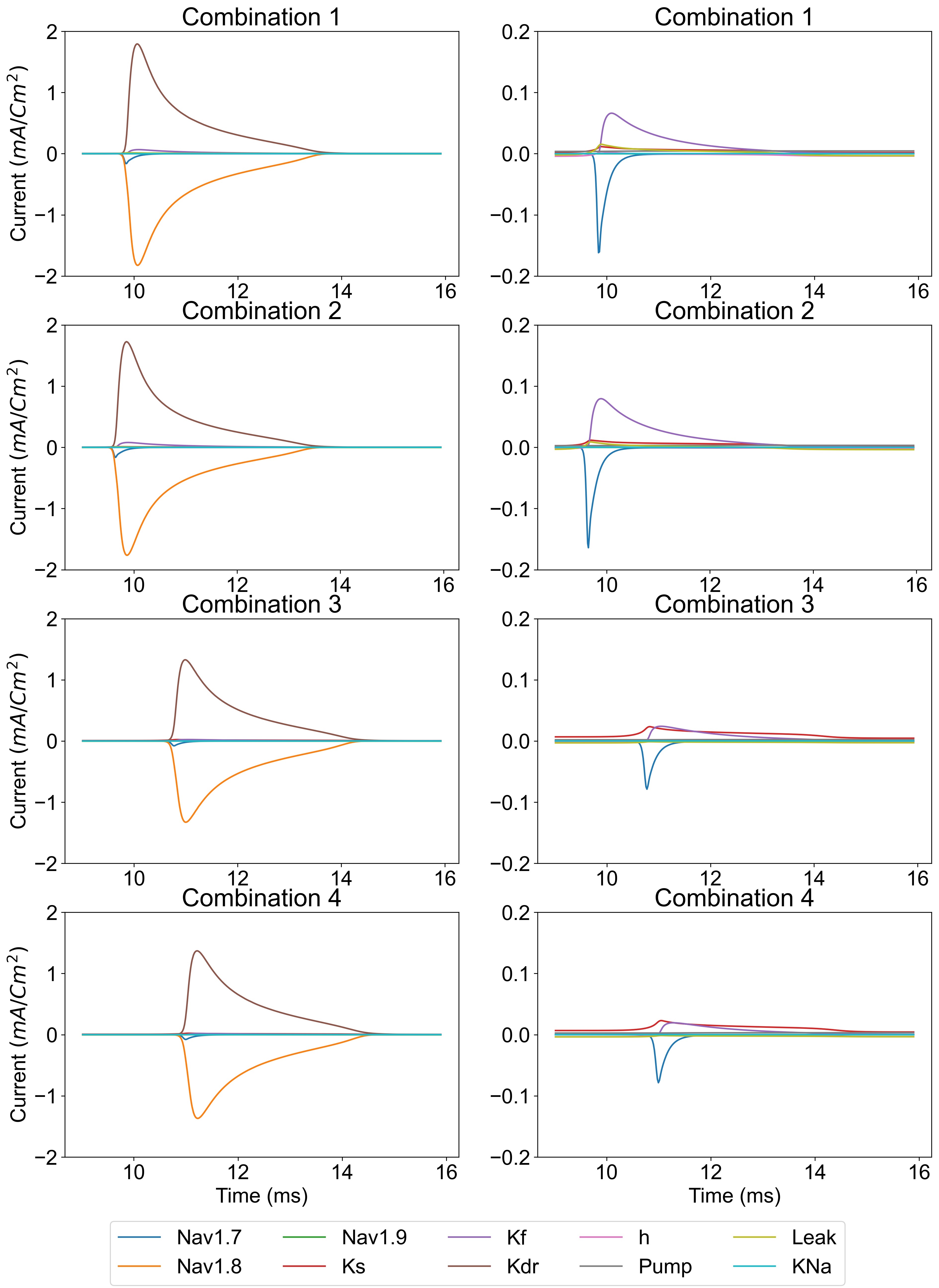

Supplement: SUPPLEMENTARY FIGURE A6 — The ionic currents during an action potential for the four final combinations of the grid search. The panels on the left show all ionic currents, the panels on the right only the small ionic currents. [file Image_6.JPEG]

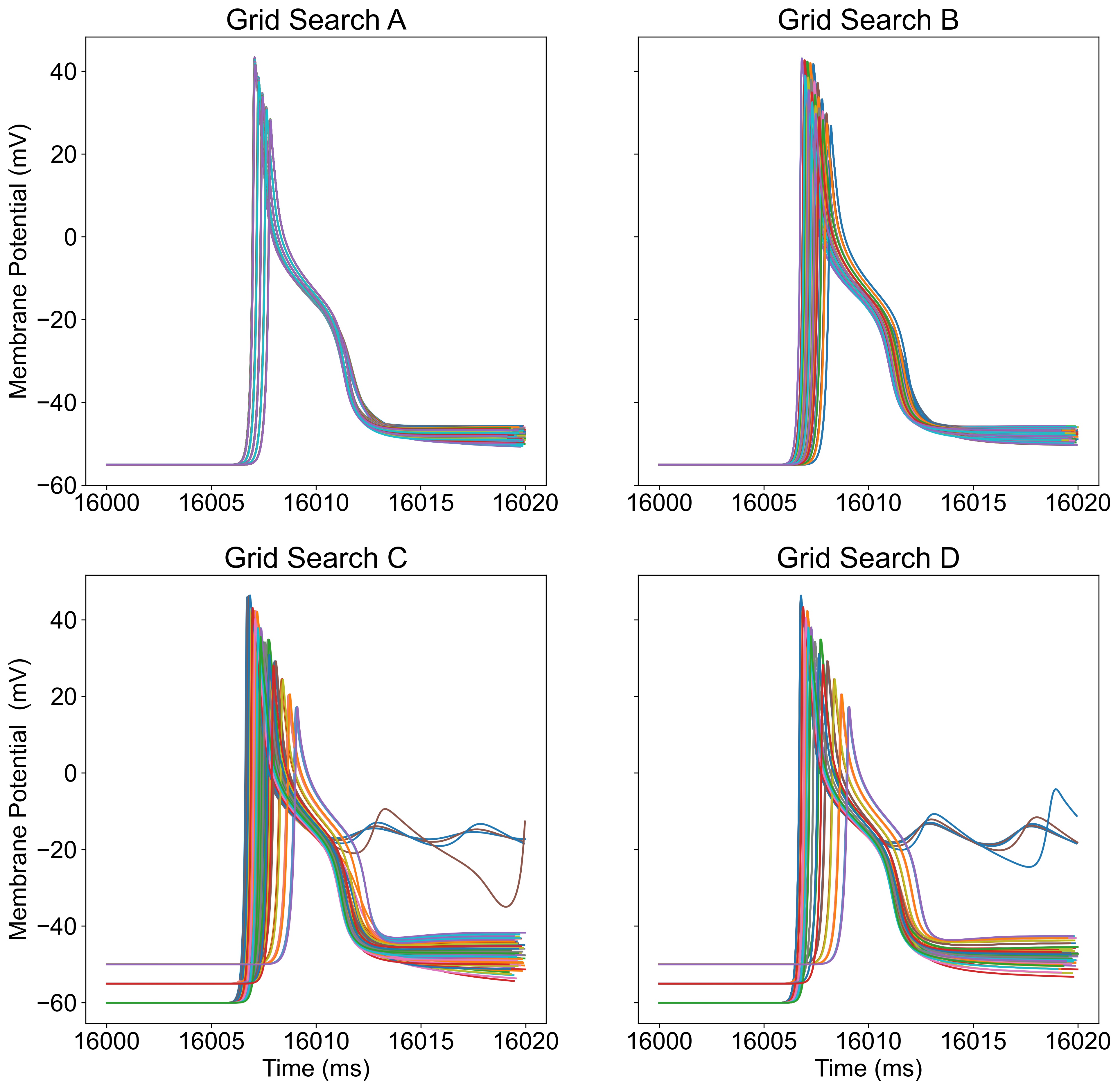

Supplement: SUPPLEMENTARY FIGURE A7 — Spike shape of all performed model evaluations. For Grid Search A (for the channels K dr , h, and K f ) and Grid Search B (for the channels K dr , h, and Na v 1.7) all spike shapes resemble qualitatively the original model, while for Grid Search C (for the channels K dr , h, and the RMP) and Grid Search D (for the channel K dr , the Na-K-pump, and the RMP) some spikes show strange behavior and were excluded from the further analysis. [file Image_7.JPEG]
